# Mechanism of Zn^2+^ regulation of cellulase production in *Trichoderma reesei* Rut-C30

**DOI:** 10.1186/s13068-023-02323-1

**Published:** 2023-04-28

**Authors:** Ni Li, Jing Li, Yumeng Chen, Yaling Shen, Dongzhi Wei, Wei Wang

**Affiliations:** The State Key Laboratory of Bioreactor Engineering, East China University of Science and Technology, 130 Meilong Road, P. O. Box 311, Shanghai, 200237 China

**Keywords:** *Trichoderma reesei*, Filamentous fungi, Zn^2+^ stimulation, Cellulase, *Plc-e*, *zafA*

## Abstract

**Background:**

*Trichoderma reesei* Rut-C30 is a hypercellulolytic mutant strain that degrades abundant sources of lignocellulosic plant biomass, yielding renewable biofuels. Although Zn^2+^ is an activator of enzymes in almost all organisms, its effects on cellulase activity in *T. reesei* have yet to be reported.

**Results:**

Although high concentrations of Zn^2+^ severely suppressed the extension of *T. reesei* mycelia, the application of 1–4 mM Zn^2+^ enhanced cellulase and xylanase production in the high-yielding cellulase-producing Rut-C30 strain of *T. reesei*. Expression of the major cellulase, xylanase, and two essential transcription activator genes (*xyr1* and *ace3*) increased in response to Zn^2+^ stimulation. Transcriptome analysis revealed that the mRNA levels of *plc-e* encoding phospholipase C, which is involved in the calcium signaling pathway, were enhanced by Zn^2+^ application. The disruption of *plc-e* abolished the cellulase-positive influence of Zn^2+^ in the early phase of induction, indicating that *plc-e* is involved in Zn^2+^-induced cellulase production. Furthermore, treatment with LaCl_3_ (a plasma membrane Ca^2+^ channel blocker) and deletion of *crz1* (calcineurin-responsive zinc finger transcription factor 1) indicated that calcium signaling is partially involved in this process. Moreover, we identified the zinc-responsive transcription factor *zafA*, the transcriptional levels of which declined in response to Zn^2+^ stress. Deletion of *zafA* indicates that this factor plays a prominent role in mediating the Zn^2+^-induced excessive production of cellulase.

**Conclusions:**

For the first time, we have demonstrated that Zn^2+^ is toxic to *T. reesei*, although promotes a marked increase in cellulase production. This positive influence of Zn^2+^ is facilitated by the *plc-e* gene and *zafA* transcription factor. These findings provide insights into the role of Zn^2+^ in *T. reesei* and the mechanisms underlying signal transduction in cellulase synthesis.

**Supplementary Information:**

The online version contains supplementary material available at 10.1186/s13068-023-02323-1.

## Background

The derivation of bioethanol from the degradation of lignocellulose is an important process [1, 2]. Saccharification, a vital step in the degradation of lignocellulose, is catalyzed by cellulases produced by filamentous fungi, such as *Trichoderma reesei* and *Aspergillus nidulans* [3–5]. Given its excellent extracellular enzyme production, safety, and reliability, *T. reesei* is used industrially as a model strain for cellulase production [6, 7]. Xyr1, a global transcriptional activator, regulates the expression of cellulase and xylanase genes [8], and a disruption of *xyr1* has been demonstrated to result in abrogation of the expression of almost all cellulase- and hemicellulase-encoding genes [9]. In 2014, a further important transcriptional activator, *ace3*, was reported to regulate the production of cellulase and xylanase [10]. Although the loss of *ace3* was found to result in slight reductions in the expression of hemicellulases [11] and xylanases [12], it completely abolished cellulase production. Microorganisms sense their surroundings and respond to external signals via a network of signal transduction pathways that provide strict control of cellulase production [13]. Among such pathways, the calcium signaling pathway is essential and highly conserved in filamentous fungi [13].

Ca^2+^ play vital cellular roles as a ubiquitous second messenger regulating cell growth, virulence, and stress resistance [14], and is a core component of the calcium signal transduction pathway in filamentous fungi [15]. In *T. reesei*, Chen et al. [16] demonstrated that Mn^2+^ regulates cellulase gene expression via calcium signaling, and Xu et al. [17, 18] have demonstrated that addition of Mn^2+^ and Na^+^ to liquid cultures of *Ganoderma lucidum* induces the biosynthesis of ganoderic acid via calcineurin signaling transduction. Subsequently, Gao et al. [19] revealed that reactive oxygen species (ROS) and Ca^2+^ cross-regulate hyphal branching and ganoderic acid biosynthesis induced by Cu^2+^ in *G. lucidum*. The complete Ca^2+^ signaling pathway includes free Ca^2+^, calmodulin (Cam), calcineurin (Cna), and calcineurin-responsive zinc-finger transcription factor 1 (Crz1/CrzA), and in response to an increase in cytoplasmic concentrations of Ca^2+^, activation of Cam and Cna promotes the dephosphorylation of Crz1/CrzA, which acts on downstream pathway genes [20].

Cytosolic Ca^2+^ levels increase via two pathways, in the first of which, Ca^2+^ from the external environment enters the cytoplasm via ion channels in the cell membrane [21–23], whereas in the second, Ca^2+^ within the intracellular Ca^2+^ pool enters the cytoplasm via a PI-PLC/IP3-mediated pathway [24]. In this latter pathway, phospholipase C (PLC) is activated in response to extracellular signals, promoting an increase in IP3 content, which subsequently leads to the release of Ca^2+^ from the intracellular Ca^2+^ pool [25, 26]. According to Chen et al. [15], *plc-e* can be activated by *N*, *N*-dimethylformamide, which promotes an increase in cytosolic Ca^2+^ in *T. reesei*.

Zn^2+^ serves as an important structural or catalytic cofactor for numerous transcription factors (TFs) and enzymes, and is accordingly essential for almost all organisms, including fungi [27, 28]. A number of studies on Zn^2+^ metabolism have focused on pathogenic fungi, such as *Candida albicans* [29], *Cryptococcus gattii* [28], and *Aspergillus fumigatus* [30], in which Zn^2+^ facilitates normal growth and plays important roles in a range physiological processes [31]. Zap1, a zinc-responsive TF, was first identified in *Saccharomyces cerevisiae*, in which it controls Zn^2+^ homeostasis and adaptive responses to Zn^2+^ deficiency [32]. Homologous to *S. cerevisiae* Zap1 is the transcriptional activator ZafA identified in *A. fumigatus* [33]. In a murine model of invasive aspergillosis, cells with loss of ZafA were found to be characterized by negligible virulence [33]. Schneider et al. demonstrated that Zap1 (an ortholog of *S. cerevisiae* Zap1) plays a vital role in the regulation of Zn^2+^ homeostasis and modulation of virulence in *C. gattii* [28]. Comparatively, however, there have been few studies that have examined the role of zinc in non-pathogenic filamentous fungi.

In this study, we used the high-yielding RUT-C30 strain of *T. reesei* as a parent strain to study the mechanisms whereby extracellular Zn^2+^ induces cellulase production. We discovered that the addition of intracellular Zn^2+^ causes a significant increase in cellulase production and has a strong inhibitory effect on hyphal growth. Transcriptome analysis and gene deletion were used to elucidate the molecular mechanisms underlying the Zn^2+^-induced cellulase production. In addition, we identified a zinc-responsive TF ZafA (an ortholog of *S. cerevisiae* Zap1 and *A. fumigatus* ZafA) in *T. reesei* and demonstrated its relevance with respect to cellulase production in response to Zn^2+^. The findings of this study provide important insights for further elucidation of the mechanisms whereby Zn^2+^ influences cellulase production in the filamentous fungi *T. reesei*.

## Results

### Effects of Zn^2+^ on hyphal growth and cellulase and xylanase production in ***T. reesei***

**Fig. 1:**
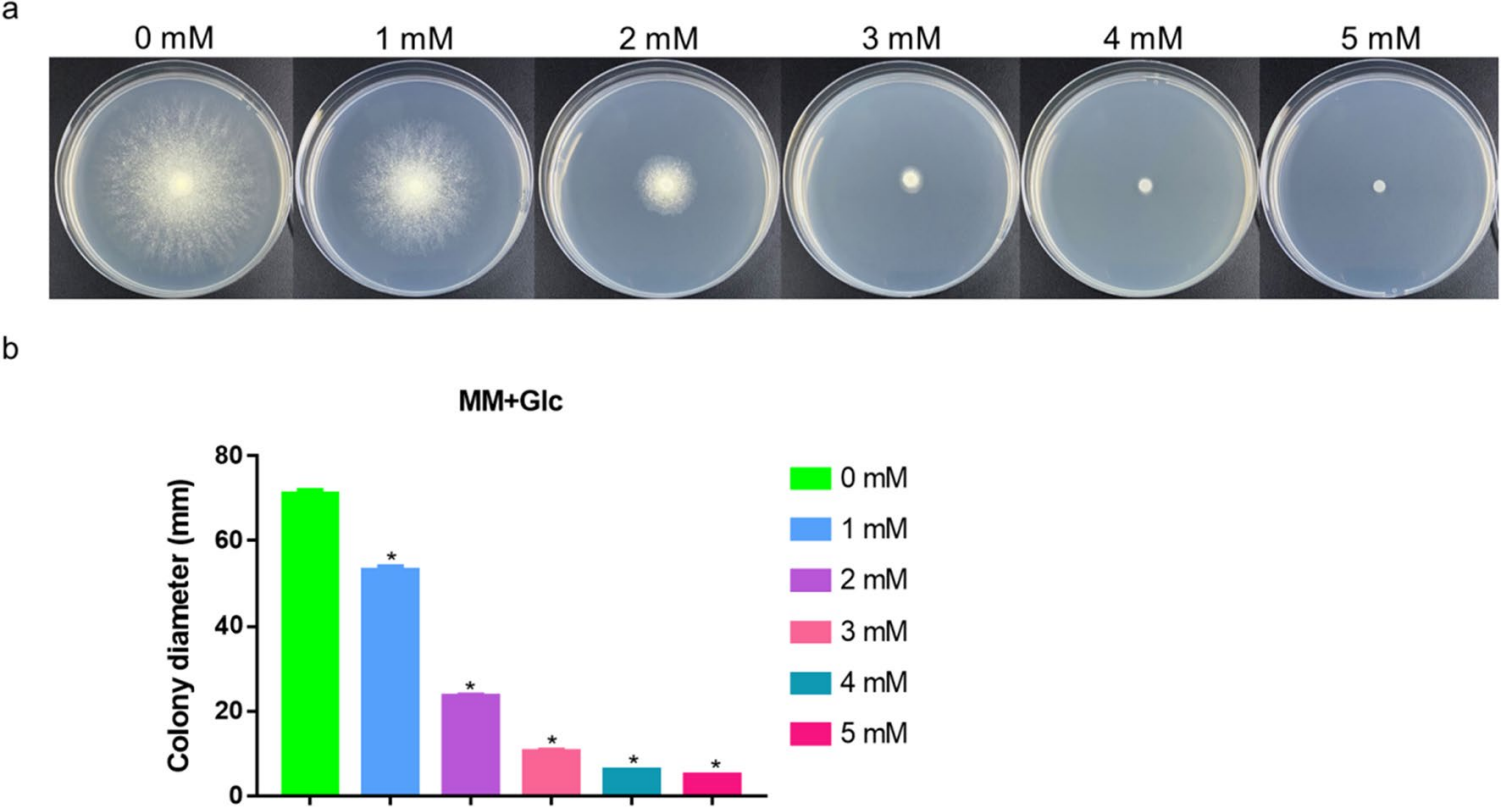
Different concentrations of Zn^2+^ influence the hyphal growth of the *Trichoderma reesei* RUT-C30 strain. **a** RUT-C30 grown on MM plates supplemented with Zn^2+^ at final concentrations of 0–5 mM. **b** Colony diameter of *T. reesei* RUT-C30 cultured on MM plates under different concentrations of Zn^2+^. The final values are presented as the means ± standard deviation (SD) of three independent experimental results. Asterisks indicate significant differences compared with the control (*P < 0.05, according to Student’s *t*-test). MM, minimal medium

To study the effect of Zn^2+^ on hyphal growth, the same amounts of fresh RUT-C30 conidia were inoculated onto minimal medium (MM) plates [supplemented with different concentrations of Zn^2+^ (0–5 mM final concentration) and 2% (w/v) glucose as the sole carbon source] for 4 days to compare colony growth. As shown in Fig. 1a, Zn^2+^ strongly inhibited hyphal growth. When the concentration of Zn^2+^ was increased to 1 mM, growth was inhibited to a certain extent compared with the untreated strain (by approximately 25.4%) (Fig. 1b). Compared with the untreated strain, treatment with 2 mM Zn^2+^ led to a 66.9% reduction in colony diameter, and treatment with ≥ 3 mM Zn^2+^ suppressed virtually all colony growth. These results accordingly revealed that at higher concentrations, Zn^2+^ represents a stressor that inhibits *T. reesei* growth.

**Fig. 2:**
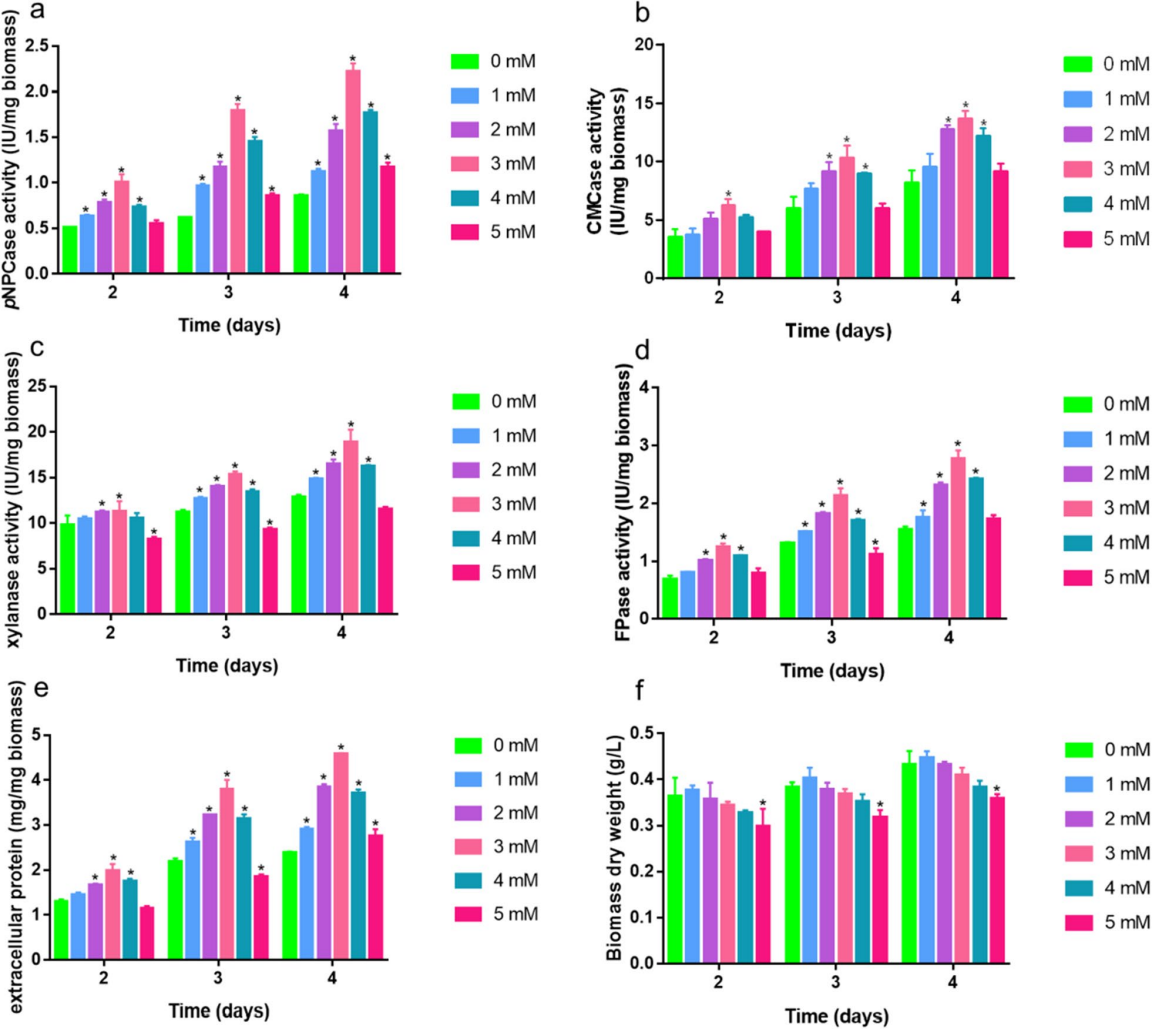
Different concentrations of Zn^2+^ influence the cellulase and xylanase synthesis of the *Trichoderma reesei* RUT-C30 strain. *p*NPCase activity (**a**), CMCase activity (**b**), xylanase activity (**c**), FPase activity (**d**), total protein concentrations (**e**), and biomass dry weight (**f**) of the RUT-C30 strain were determined after culturing for 2, 3, or 4 days in liquid MM containing different concentrations of Zn^2+^ (0–5 mM) and 1% (w/v) Avicel as the sole carbon source. The final values are presented as the means ± standard deviation (SD) of the three independent experimental results. Asterisks indicate significant differences compared with the control (*P < 0.05, according to Student’s* t*-test). MM, minimal medium

Given the effects of Zn^2+^ on the growth of strains, we adopted the transfer method in this study. Equal masses of *T. reesei* RUT-C30 pre-cultured mycelia were transferred to MM supplemented with 1% (w/v) Avicel as the sole carbon and different concentrations of Zn^2+^ (0–5 mM) and incubated at 28℃ for 4 days to assess the effect of Zn^2+^ treatment on the production of cellulase, xylanase, and extracellular protein. As shown in Fig. 2, the addition of 1–4 mM Zn^2+^ promoted marked enhancements of cellulase, xylanase and extracellular protein per gram of *T. reesei* mycelium, annulling its negative effects on growth. Supplementation with 3 mM Zn^2+^ was found to have the most pronounced effects in this regard. Specifically, the addition of 3 mM Zn^2+^ stimulated *p*NPCase activity (representing *exo*-β-glucanase activity), which was enhanced by approximately 96.5% to 191.3% compared with the control without 3 mM Zn^2+^ supplementation (Fig. 2a). As shown in Fig. 2b, 3 mM Zn^2+^ also promoted a significant increase in CMCase activity (representing *endo*-β-glucanase activity), by approximately 67.9% to 77.7% compared with that of the control. Furthermore, compared with the control, supplementation with 3 mM Zn^2+^ resulted in approximate 46.9% and 82.6% increases in xylanase and filter paper hydrolase activities (FPase, representing total extracellular cellulase activity), respectively (Fig. 2c, d). As illustrated in Fig. 2e, we detected an approximate 92.8% increase in extracellular protein concentration in response to supplementation with 3 mM Zn^2+^ compared with the control strain. In contrast, exposure to 5 mM Zn^2+^ was found to have negative effects on CMCase, xylanase, and FPase activities, which we speculate could be attributed to the severe growth inhibition effects at high concentrations (Fig. 2f). Accordingly, in further studies, 3 mM Zn^2+^ was selected as the optimal concentration of for enhancing cellulase yields.

**Fig. 3:**
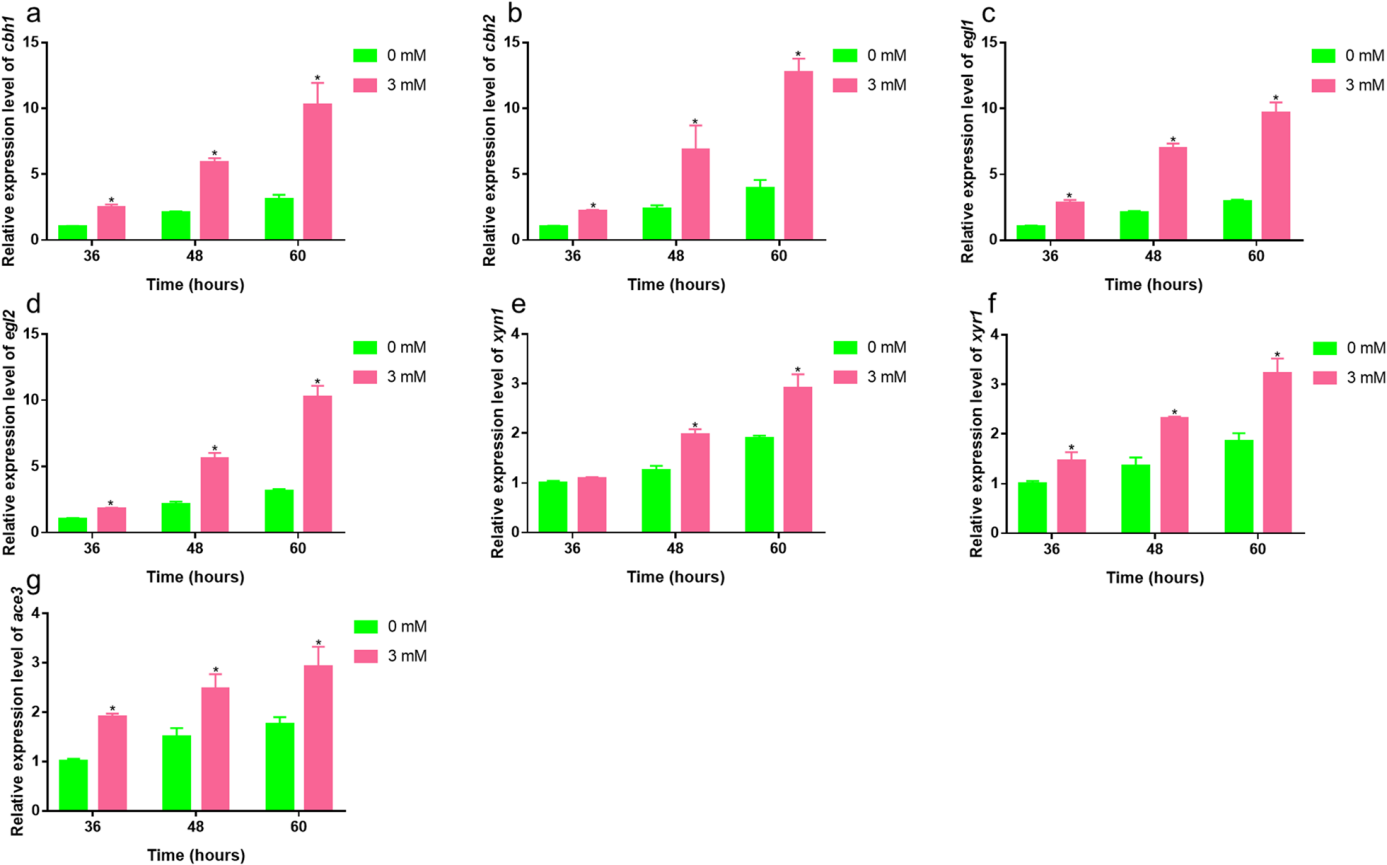
Effects of 3 mM Zn^2+^ on cellulase-related gene transcription levels in the *Trichoderma reesei* RUT-C30 strain. Transcriptional levels of *cbh1* (**a**), *cbh2* (**b**), *egl1* (**c**), *egl2* (**d**), *xyn1* (**e**), *xyr1* (**f**), and *ace3* (**g**) of the RUT-C30 strain were detected after culturing for 36, 48 or 60 h in liquid MM containing 0 or 3 mM Zn^2+^ with 1% (w/v) Avicel as the sole carbon source. The final values are presented as the means ± standard deviation (SD) of three independent experimental results. Asterisks indicate significant differences compared with the control (*P < 0.05, according to Student’s* t*-test). MM, minimal medium

To further investigate the effects of Zn^2+^ on cellulase and xylanase production, we determined the transcription levels of the four main cellulase genes (*cbh1*, *cbh2*, *egl1*, and *egl2*), one major xylanase gene (*xyn1*), and two essential transcriptional activators of cellulase and xylanase (*xyr1* and *ace3*) using real-time quantitative PCR (RT-qPCR). Consistent with the aforementioned cellulase and extracellular protein results, we found that the addition of 3 mM Zn^2+^ promoted marked increases in the expression levels of the four main cellulase genes and one major xylanase gene by approximately 0.54- to 2.33-fold compared with the control at 60 h (Fig. 3a–e). Consistently, we detected a marked up-regulation of the transcriptional levels of *xyr1* and *ace3* at all assessed time points (Fig. 3f, g).

To the best of our knowledge, this is the first time that Zn^2+^ supplementation has been demonstrated to promote a significant increase in the cellulase and xylanase production of *T. reesei* Rut-C30. However, the specific induction mechanisms have yet to be sufficiently established and accordingly warrants further investigation.

### Transcriptomic changes in *T. reesei* following exposure to Zn^2+^

To further determine how Zn^2+^ influences *T. reesei* at the transcriptional level, we performed whole-transcriptome shotgun sequencing (RNA-seq) using RUT-C30 cultured for 48 h in MM containing 0 or 3 mM Zn^2+^, with 1% Avicel as the sole carbon source. We performed Illumina NovaSeq6000 RNA sequencing to analyze three independent biological samples from each of the assessed conditions. Following sequence quality control (41,388,676–54,546,188 reads, with no significant difference between parallel biological samples), the sequences of the total reads were mapped to the latest *T. reesei* reference genome (https://www.ncbi.nlm.nih.gov/assembly/GCA_002006585.1) with a coverage of 94.82–95.42%. The results of Pearson correlation analysis (r^2^ ≥ 0.718) revealed that there was a strong correlation between the three biological replicates of the strain with or without 3 mM Zn^2+^ (Additional file 1: Fig. S1). Subsequently, we screened genes for differential expression between the two conditions based on the following thresholds: a Log_2_fold change (Log_2_fc) ≥ 1 and an adjected *p*-value < 0.05.

**Fig. 4:**
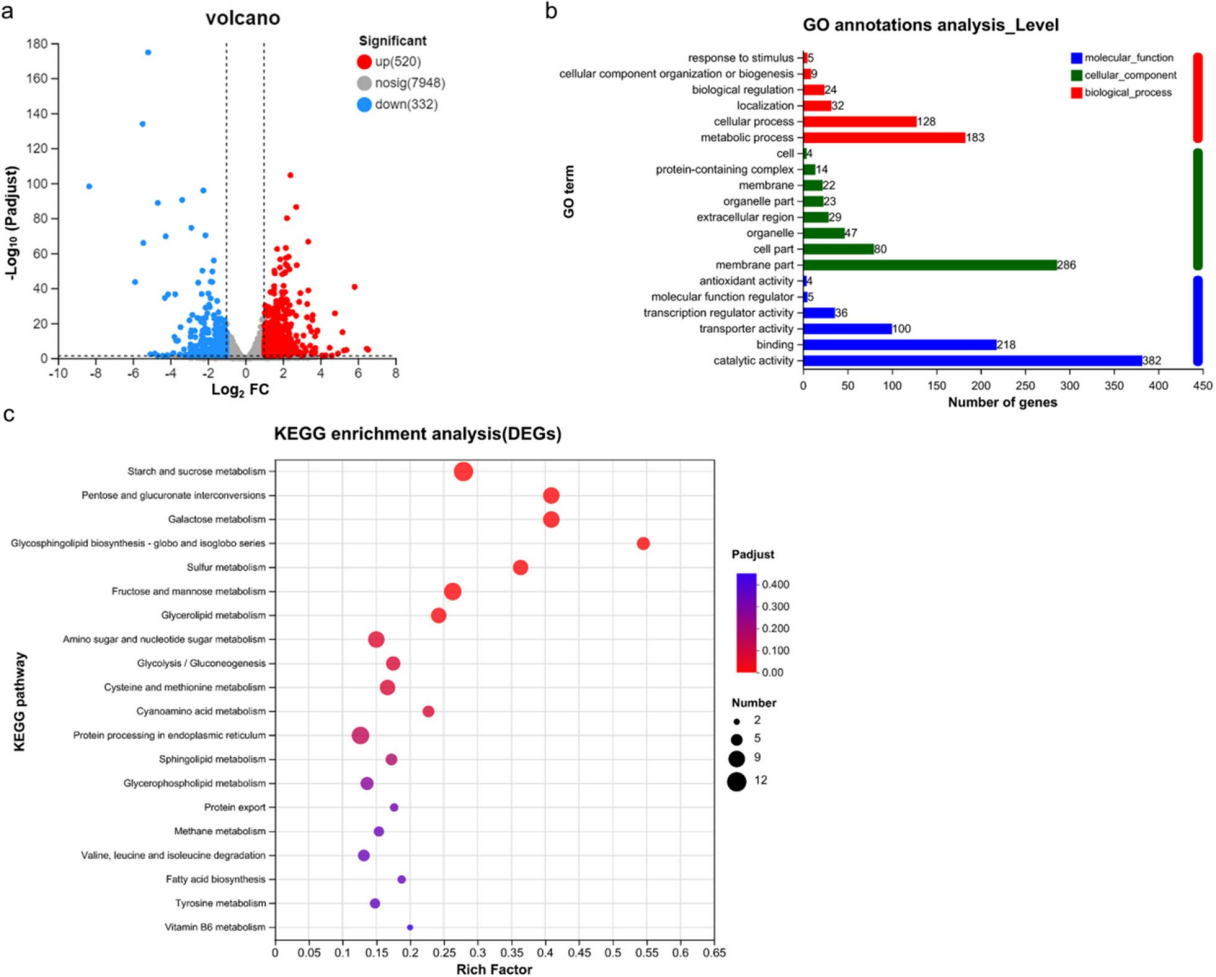
RNA‑seq analysis of the *Trichoderma reesei* RUT-C30 strain treated with 0 or 3 mM Zn^2+^. Volcano analysis (**a**), Gene ontology (GO) annotation analysis (**b**), and Kyoto Encyclopedia of Genes and Genomes (KEGG) enrichment analysis (**c**) of DEGs in the RUT-C30 strain treated with 0 and 3 mM Zn^2+^

As shown in the volcano plot presented in Fig. 4, in the presence of 3 mM Zn^2+^, 852 genes were differentially expressed, of which 520 were upregulated and 332 were downregulated (3 mM_vs._0 mM; Fig. 4a). Gene ontology (GO) annotation analysis of these differentially expressed genes (DEGs) revealed that the most enriched genes are associated with catalytic activity, membrane part, binding, and metabolic processes (Fig. 4b). A histogram representing the findings of Kyoto Encyclopedia of Genes and Genomes (KEGG) analysis revealed that the most enriched pathways affected by Zn^2+^ included “starch and sucrose metabolism”, “fructose and mannose metabolism,” “protein processing in endoplasmic reticulum,” “galactose metabolism,” “pentose and glucuronate interconversions,” and “amino sugar and nucleotide sugar metabolism” (Fig. 4c). Collectively, the data indicated that in the presence of Zn^2+^, significant changes occurred in multiple signaling pathways, which are worthy of further investigation.

Among the 34 genes associated with cellulase and hemicellulose degradation in *T. reesei*, we detected upregulated transcriptional levels in 28 genes and downregulated levels in two (M419DRAFT_ 124931 and M419DRAFT_103113) (Table 1). Notably, we detected marked increases (Log_2_fc ≥ 2) in the mRNA levels of two main cellobiohydrolases (CEL7A and CEL6A), two endoglucanases (CEL7B and CEL45A), one cellulose-binding protein CIP1 (M419DRAFT_121449) [34], swollenin (M419DRAFT_104220) [35], one xylanase (XYN3), mannan endo-1,4-beta-mannosidase MAN1 (M419DRAFT_122377), and alpha-galactosidase AGL3 (M419DRAFT_39277). Additionally, 15 TFs are known to be associated with cellulase and hemicellulase expression, of which three are positive transcription regulators with increased mRNA levels (*xyr1*, *ace3*, and *vib1* [37]) and one is a negative transcription regulator with reduced mRNA levels (*rce1* [36]) (Table 2)*.* These results are consistent with the marked increases in *p*NPCase, CMCase, xylanase, and FPase activities and RT-qPCR data for RUT-C30 cells treated with 3 mM Zn^2+^.

**Table 1:** Comparison of cellulase and hemicellulase gene expression levels in the *Trichoderma reesei* RUT-C30 strain grown with or without 3 mM Zn^2+^

Gene ID	Description	Log_2_fc	Adjusted* p*	Up/down
*Cellulose degradation-related genes*				
125125	Cellobiohydrolase CBH1/CEL7A	2.222487007	1.0103E−80	Up
122470	Cellobiohydrolase CBH2/CEL6A	2.309858257	9.90278E−59	Up
5304	Endoglucanase EGL1/CEL7B	2.251992253	1.78524E−53	Up
72489	Endoglucanase EGL2/CEL5A	1.995739374	2.76844E−37	Up
124438	Endoglucanase EGL3/CEL12A	1.574290802	2.22816E−15	Up
139633	Endoglucanase EGL4/CEL61A	1.858709256	3.75968E−57	Up
25940	Endoglucanase EGL5/CEL45A	2.161014286	5.68478E−58	Up
127115	Beta-glucosidase BGL2/CEL1A	0.673994855	3.30009E−10	Slightly up
134448	Alpha-glucosidase	1.350744648	2.19791E−06	Up
125268	Beta-glucosidase CEL3C	0.2609728	0.373108278	NS
121449	Cellulose-binding protein CIP1	2.042626642	4.91937E-42	Up
125575	Cellulose-binding protein CIP2	1.382394269	2.45461E−06	Up
104220	Swollenin	2.165213146	9.85563E−64	Up
136547	Cel3a	1.401993758	5.077E−14	Up
77989	Putative beta-glucosidase CELB	0.376282586	0.002632392	Slightly up
122639	Beta-glucosidase CEL3D	1.554885912	6.6498E−10	Up
76309	Alpha-glucosidase	0.713796204	0.034331223	Slightly up
136825	Alpha-glucosidase GLS2	− 0.214375838	0.409158437	NS
98040	Alpha-glucosidase	0.449617497	1.64398E−05	Slightly up
142027	Beta-glycosidase	0.010759773	0.964165236	NS
131175	Alpha-1/3-glucanase	1.278426506	0.173351439	NS
*Hemicellulose degradation-related genes*				
38418	Xylanase XYN1	1.056916586	5.4607E−08	Up
124931	Xylanase XYN2	− 1.024513995	0.001577744	Down
23616	Xylanase XYN3	2.445893508	3.94175E−20	Up
90847	Xylanase XYN4	0.836038281	6.21109E−11	Slightly up
140746	Beta-xylosidase BXL1	0.735646464	1.45211E−08	slightly up
139631	Acetyl xylan esterase AXE1	1.459031237	2.06915E−11	Up
122377	Mannan endo-1,4-beta-mannosidase MAN1	2.059384787	4.7073E−28	Up
71638	Alpha-galactosidase	1.196530627	4.90202E−30	Up
90302	Alpha-glucuronidase GLR1	1.391700754	3.23708E−12	Up
39277	Alpha-galactosidase AGL3	2.409734976	2.9746E−105	Up
126869	Alpha-1,6-mannanase	1.857455587	1.05375E−23	Up
103113	Alpha-1,6-mannanase	− 0.477886119	0.000137425	Slightly down
91133	Alpha-galactosidase	1.350858721	0.004818377	Up

*NS* not significant

adjusted *p* > 0.05; the gene ID of RUT-C30 is used in the table

**Table 2:** Comparison of transcription factor gene expression levels in the *Trichoderma reesei* RUT-C30 strain grown with or without 3 mM Zn^2+^

Transcription factor genes	Gene ID	Log_2_fc	Positive/negative-acting	Adjusted *p*	Up/Down
Xyr1	98788	1.533791051	Positive	5.72702E−42	Up
Ace3	98455	1.120730286	Positive	5.40208E−21	Up
Vib1	125610	1.919072808	Positive	2.81E−33	Up
Ace1	122363	− 0.398721897	Negative	0.069669905	NS
Ace2	32395	0.619714266	Positive	0.000517735	slightly up
Ace4	36511	− 0.104047118	Positive	0.569481038	NS
Clr-1	68701	0.714993818	Positive	0.000295961	slightly up
Clr-2	76250	0.391743296	Positive	0.077901826	NS
BglR	91236	0.461942603	Positive	0.000834524	slightly up
Hap2	93466	0.095873666	Positive	0.734294215	NS
Hap3	24298	0.241225909	Positive	0.115847056	NS
AreA	140814	0.589879921	Positive	6.8502E−05	slightly up
Rce1	6520	− 1.505078796	Negative	2.84E−18	Down
Ctf1	10530	− 0.164406285	Negative	0.321431961	NS
Rce2	109517	0.221815641	Negative	0.183986109	NS

*NS* not significant

Adjusted *p* > 0.05; the gene ID of RUT-C30 is used in the table

### PLC-E is required for Zn^2+^ induction of cellulase production

Metal ions have been reported to regulate cellulase gene expression via calcium signaling in *T. reesei* [16], and consequently, we speculated as to whether calcium signaling would be involved in Zn^2+^-induced cellulase expression. To verify this conjecture, we determined the mRNA levels of four major calcium signal pathway-related genes based on transcriptional profiling (Table 3). Notably, we detected a significant increase in the mRNA levels of *plc-e* encoding a phospholipase C protein, which can be activated by extracellular receptors, and induces the release of calcium from internal stores via the generation of inositol-1,4,5-trisphosphate (IP3) [37].

**Table 3:** Comparison of calcium signal transduction-related genes expression levels in *Trichoderma reesei* RUT-C30 strain with or without 3 mM Zn^2+^

Gene ID	Description	Log_2_fc	Adjusted* p*	Up/down
139,407	*plc-e*	1.949727396	1.8149E-38	Up
113,721	*cam*	0.466481553	0.000660264	Slightly up
136,884	*cna1*	0.01591147	0.947533072	NS
141,391	*crz1*	0.463634306	0.04692323	Slightly up

*NS* not significant

Adjusted *p* > 0.05; the gene ID of RUT-C30 is used in the table

**Fig. 5:**
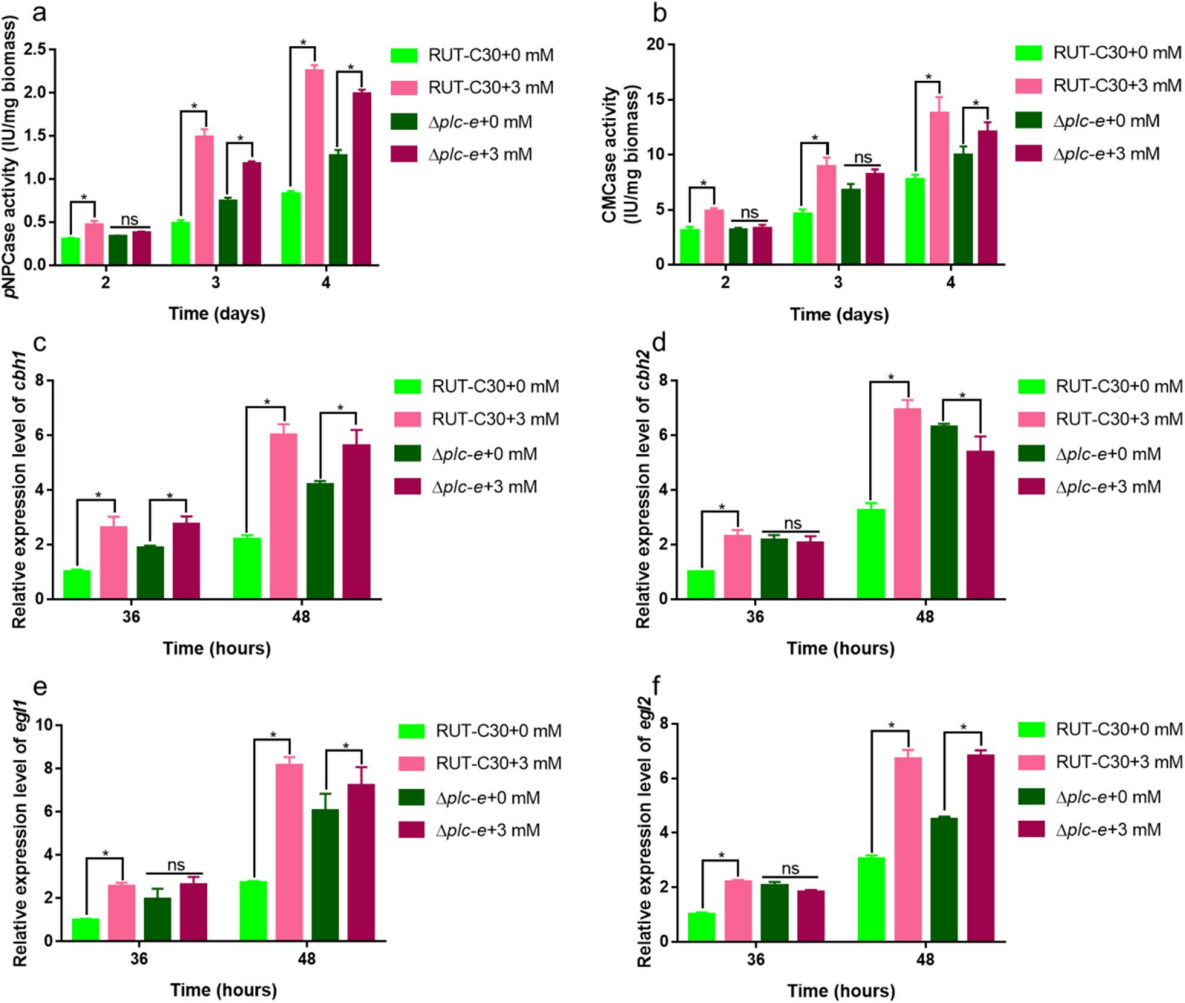
Effects of *plc-e* on cellulase production after Zn^2+^ addition. *p*NPCase activity (**a**), CMCase activity (**b**) of *Trichoderma reesei* RUT-C30 and Δ*plc-e* cultured for 2, 3, or 4 days in liquid MM with or without 3 mM Zn^2+^ and 1% (w/v) Avicel as the sole carbon source, respectively. The mRNA levels of *cbh1* (**c**), *cbh2* (**d**), *egl1* (**e**), and *egl2* (**f**) were also detected. The final values are presented as the means ± standard deviation (SD) of three independent experimental results. Asterisks indicate significant differences compared with the control (*P < 0.05, according to Student’s *t*-test). MM, minimal medium

Furthermore, to determine whether *plc-e* plays an important role in Zn^2+^-induced excessive production of cellulase, *plc-e* was deleted in *T. reesei* RUT-C30 to obtain the mutant strain Δ*plc-e*. Interestingly, the facilitation effect of Zn^2+^ on cellulase synthesis in Δ*plc-e* was initially suppressed during the early phase (36 h for qPCR and 2 days for activities) and effectively attenuated in the latter phase (Fig. 5a–f), thereby providing evidence to indicate that *plc-e* is involved in the Zn^2+^ induction process. In the absence of Zn^2+^, the loss of *plc-e* promoted a slight enhancement of *p*NPCase and CMCase activities, whereas in the presence of Zn^2+^, we detected reductions in *p*NPCase and CMCase activities in Δ*plc-e* compared with those in the parental RUT-C30 (Fig. 5a, b). Collectively, these findings indicate that the deletion of *plc-e* effectively attenuates the induction effect of Zn^2+^ on cellulase production. Similar findings were obtained with respect to the transcriptional levels of *cbh1, cbh2, egl1,* and *egl2* (Fig. 5c–f).

On the basis of transcription profiling, we established that the transcriptional levels of the calcium signaling genes (*cam* and *crz1*) were slightly enhanced in the presence of Zn^2+^ (Table 3). RT-qPCR was used to quantitatively determine differences in the expression levels of these genes in the presence and absence of 3 mM Zn^2+^. As shown in Additional file 2: Fig. S2, the transcriptional levels of *cam* were slightly enhanced after 36 h of treatment with Zn^2+^.

To evaluate whether calcium signal transduction plays a role in the Zn^2+^ induction process, we used LaCl_3_ (a plasma membrane Ca^2+^ channel blocker) to inhibit the influx of external Ca^2+^ [38], and we generated the *crz1* deletion strain in RUT-C30 to inhibit the calcium signal transduction pathway. Analyses of cellulase activities (*p*NPCase and CMCase activities) and transcriptional levels of key cellulase genes (*cbh1* and *egl1*) revealed that treatment with LaCl_3_ led to a marked reduction in cellulase activity and the transcriptional levels of *cbh1* and *egl1* compared with those in the no-LaCl_3_ control, regardless of the presence of Zn^2+^ (Additional file 3: Fig. S3). Furthermore, in the presence of LaCl_3_, the facilitation effect of Zn^2+^ on cellulase synthesis was significantly attenuated during the early phase of induction (2 to 3 days) (Additional file 3: Fig. S3a, b). Quantitative analysis using RT-qPCR revealed that exposure to LaCl_3_ had a slightly negative effect on Zn^2+^-induced *cbh1* and *egl1* expression (Additional file 3: Fig. S3c, d). Collectively, these findings wound tend to indicate that calcium signal transduction is partially involved during the early phase of the Zn^2+^ induction process.

Although the calcium signaling pathway was blocked in the *crz1* deletion strain, we found that supplementation with 3 mM Zn^2+^ could still enhance the *p*NPCase and CMCase activities in this strain compared to those observed in the control (without 3 mM Zn^2+^) on day 4 (Additional file 4: Fig. S4a, b). Similar results were obtained in the RT-qPCR analysis (Additional file 4: Fig. S4c, d), thus indicating that in addition to the calcium signaling pathway, other pathways are involved in Zn^2+^-induced cellulase synthesis.

### Identification of the zinc-responsive transcription factor *zafA*

In fungi, zinc-responsive TFs play important roles in regulating zinc homeostasis [32, 33]. In this regard, the TF *zafA* has been extensively studied in *S. cerevisiae* [39], *C. gattii* [28], and *A. fumigatus* [33]. NCBI BLAST analysis revealed that M419DRAFT_96242 detected in *T. reesei* in the present study is a homolog of the *zafA* in *S. cerevisiae* and *A. fumigatus.* Furthermore, RNA‑seq analysis revealed a slight, although significant, reduction in the mRNA levels of M419DRAFT_96242 (*zafA*) following exposure to 3 mM Zn^2+^ compared with the control group (0 mM Zn^2+^) (Additional file 5: Table S1). As revealed by RT-qPCR, compared with the control, the transcription levels of M419DRAFT_96242 (*zafA*) were downregulated by 48.99% in response to 3 mM Zn^2+^ supplementation (Additional file 6: Fig. S5).

**Fig. 6:**
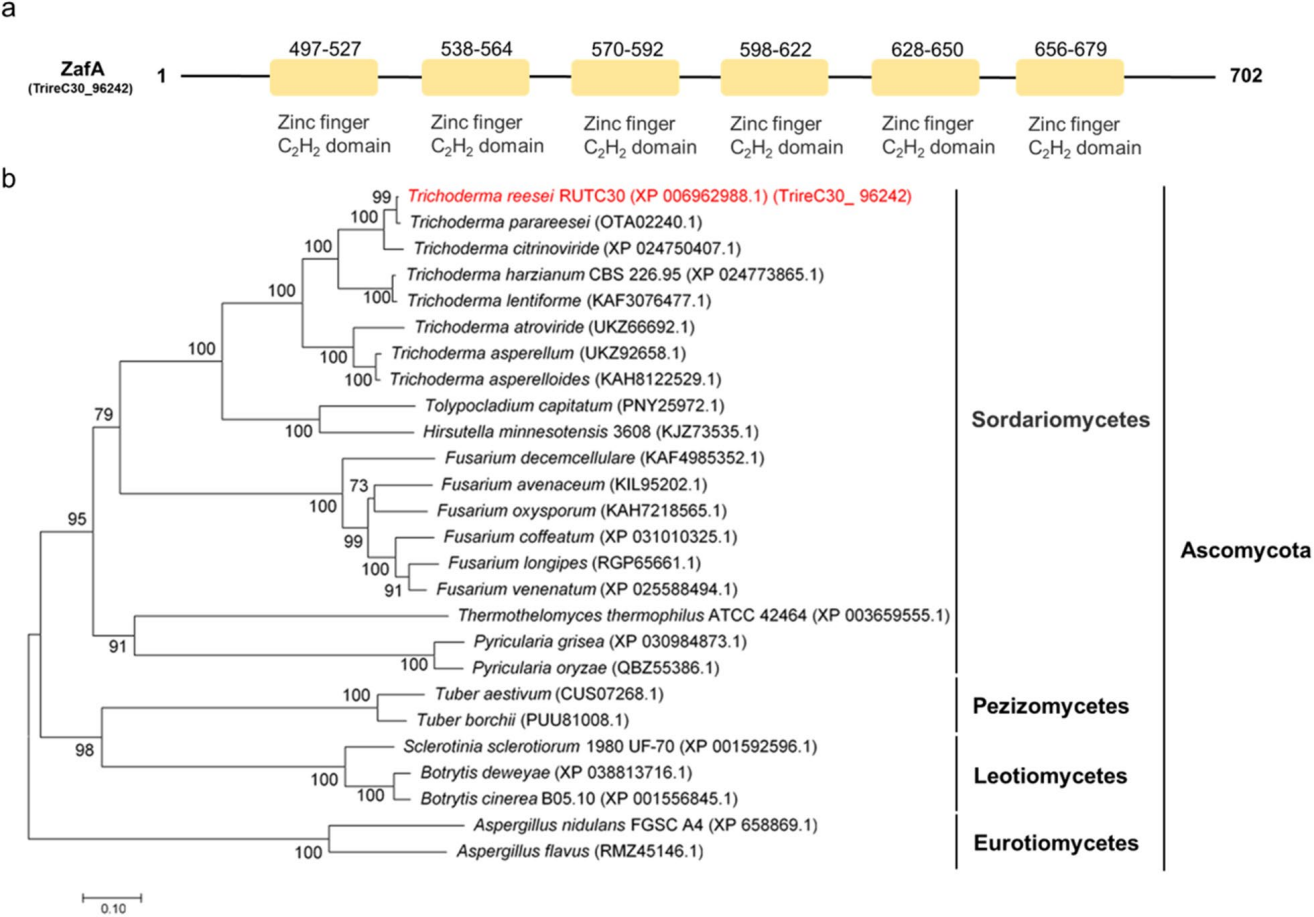
The identification of ZafA. Functional domain prediction (**a**) and phylogenetic tree analysis (**b**) of *zafA*

As shown in Fig. 6a, six zinc-finger C_2_H_2_ domains containing 702 amino acids were predicted in *zafA* using the Pfam database (http://pfam.xfam.org)*.* Phylogenetic analysis (Fig. 6b) based on the *zafA* protein sequence revealed that *zafA* homologs are widely distributed in a range of Ascomycota, including *Sordariomycetes*, *Pezizomycetes*, *Leotiomycetes*, and *Eurotiomycetes*, with high amino acid similarity. In addition, *zafA* homologs have been identified in numerous *Trichoderma* species, indicating that this protein may have a conserved function. However, the roles of *zafA* in strain growth and cellulase production (in either *T. reesei* or other cellulose-degrading species) under conditions of zinc stress have yet to be evaluated.

### ZafA mediates Zn^2+^‑stimulated excessive production of cellulase in the RUT-C30 strain

**Fig. 7:**
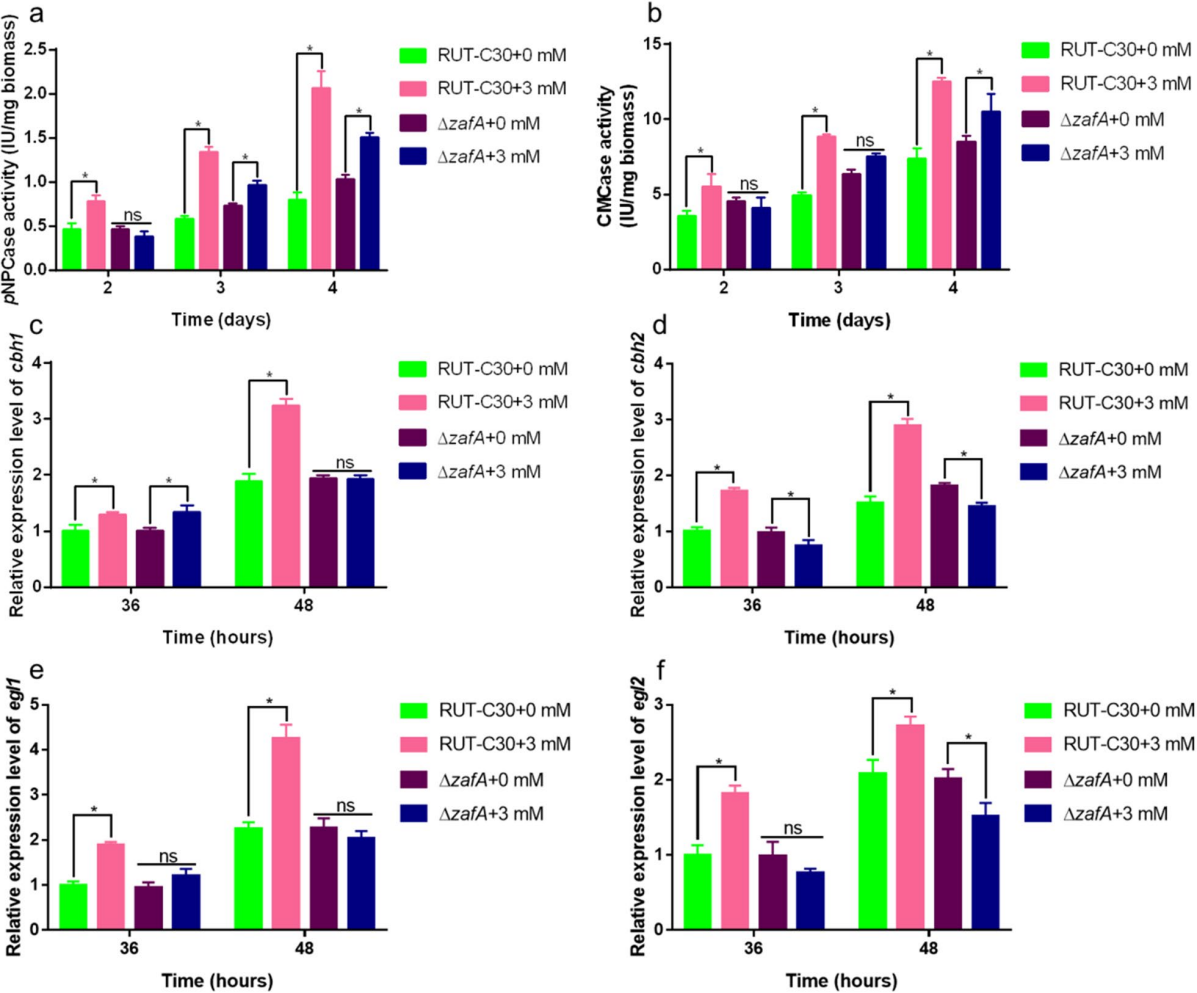
Effect of *zafA* on cellulase production after Zn^2+^ addition. *p*NPCase activity (**a**), CMCase activity (**b**) of *Trichoderma reesei* RUT-C30 and Δ*zafA* cultured for 2, 3, or 4 days in liquid MM with or without 3 mM Zn^2+^ and 1% (w/v) Avicel as the sole carbon source, respectively. The expression levels of *cbh1* (**c**), *cbh2* (**d**), *egl1* (**e**), and *egl2* (**f**) were also determined. The final values are presented as the means ± standard deviation (SD) of three independent experimental results. Asterisks indicate significant differences compared to the control (*P < 0.05, according to Student’s *t*-test). MM, minimal medium

To determine whether the reduced expression of *zafA* is associated with the Zn^2+^ induction process, we generated a *zafA* deletion strain (∆*zafA*) in which the signal transduction pathway was blocked. We investigated the effects of the parental strain RUT-C30 and ∆*zafA* mutants on cellulase production in response to 0 and 3 mM Zn^2+^ treatments. As shown in Fig. 7a, b, the deletion of *zafA* resulted in a marked reduction or complete inhibition of Zn^2+^-induced cellulase production compared with the control. In the absence of 3 mM Zn^2+^ supplementation, we detected no clear differences between the two strains with respect to *p*NPCase and CMCase activities. Compared with the parental strain RUT-C30 in the absence of 3 mM Zn^2+^, we detected marked enhancements of approximately 160.4% and 70.4% in *p*NPCase and CMCase activities, respectively, following exposure to 3 mM Zn^2+^. However, with the deletion of *zafA*, we detected notably less pronounced increases in *p*NPCase and CMCase activities of 46.4% and 23.4%, respectively, following exposure to Zn^2+^ pressure compared with no Zn^2+^ supplementation. Additionally, RT-qPCR was performed to determine the transcription levels of four major cellulase-encoding genes (*cbh1*, *cbh2*, *egl1*, and *egl2*) in *T. reesei* RUT-C30 and ∆*zafA*, and we accordingly found the transcript levels to be consistent with the cellulase activity data. The significant enhancement of the transcriptional levels of these four cellulase genes induced by Zn^2+^ was effectively attenuated by the deletion of *zafA* (Fig. 7c–f).

In summary, these findings indicate that Zn^2+^ induces an enhancement of cellulase production in *T. reesei* Rut-C30 primarily via *zafA*, and this enhancement effect induced by Zn^2+^ was effectively prevented in the ∆*zafA* mutant, in which the associated signaling pathway was blocked.

### DEGs associated with G-protein-coupled receptors and transporters

The transfer of extracellular signals to intracellular sites generally requires transport mediated by G-protein-coupled receptors (GPCRs) [40] and transporters [41]. To date 58 GPCRs have been reported in *T. reesei* [42], of which we detected the up- and downregulation of 14 and three, respectively, in response to treatments in the present study (Additional file 7: Table S2). These 17 differentially expressed GPCRs included one class III GPCR, one class V GPCR, two class VI GPCR, one class VII GPCR, one class XI GPCR, and 11 PTH11-like GPCRs, among which, 10 of the 11 PTH11-like GPCRs were downregulated, the exception being M419DRAFT_76201. In addition, among 152 transporters, we detected 44 differentially expressed DEGs, among which 31 and 13 were up- and downregulated genes, respectively (Additional file 8: Table S3). The *stp1* (M419DRAFT_136988) gene, which was notably upregulated by 2^1.18^ (2.27)-fold, has been established to be associated with cellobiose transport [43]. In addition, we identified one Fe^2+^/Zn^2+^-regulated transporter (M419DRAFT_91910) that was markedly downregulated by 2^4.29^ (19.56)-fold.

Collectively, these findings provide convincing evidence to indicate that 3 mM Zn^2+^ promotes notable changes in transcription and signal transduction in *T. reesei*.

## Discussion

In this study, we sought to examine the effects of Zn^2+^ on the growth, enzyme production, and regulatory signaling pathways of *T. reesei*. Although Zn^2+^, a key cofactor of numerous TFs and enzymes, has been widely studies in filamentous fungi [30], yeasts (*S. cerevisiae*) [39, 44], plants, and animals [45], to the best of our knowledge, there have been no published studies on the properties of Zn^2+^ in *T. reesei*. In this study, we found that strain growth on solid medium was substantially inhibited in the presence of > 2 mM Zn^2+^ (Fig. 1), thereby indicating that *T. reesei* was subjected to heightened stress. In *S. cerevisiae*, excessive Zn^2+^ has been found to cause oxidative damage to cells by regulating the expression of antioxidant defense genes [27]. In *T. reesei*, it has been found that in response increases in the concentrations of Ca^2+^, Mn^2+^, and Sr^2+^ to 100 mM, 40 mM, and 120 mM, respectively, the strain growth is generally slow and sparse [16, 20, 46]. In *Ganoderma lucidum*, hyphal branch length and growth have been observed to be significantly reduced in the presence of 5 mM Cu^2+^ [19], whereas treatment with 17 mM Ca^2+^ was found to result in a substantial reduction in the diameter of *Penicillium brevicompactum* colonies [49]. The findings of these studies indicate that fungi have specific tolerance to metal ions.

We found that exposure of *T. reesei* to Zn^2+^ promoted increases in production of the enzymes cellulase and xylanase, with maximal enhancement being recorded in those fungi treated with 3 mM Zn^2+^, a finding which would be beneficial from the perspective of industrial production (Fig. 2). In this regard, recent studies have demonstrated that extracellular supplementation with metal ions can promote the production of primary and secondary metabolites in filamentous fungi [16–19, 46]. On the basis of transcriptome analyses, we discovered that 852 genes were differentially expressed in *T. reesei* treated with 3 mM Zn^2+^, including a number of upregulated cellulase- and hemicellulose-associated genes (3 mM_vs._0 mM; Fig. 4a and Table 1), which is consistent with our RT-qPCR data (Fig. 3). Importantly, we found that the transcription levels of intracellular *plc-e* were significantly elevated in response to stimulation with Zn^2+^, whereas the deletion of *plc-e* from the parental strain (Rut-C30) resulted in an attenuation of Zn^2+^-induced cellulase production during the early phase of the Zn^2+^ induction process (Fig. 5). Previous studies have shown that *plc-e* is involved in regulating the expression of cellulase and Ca^2+^ signaling genes in *T. reesei* under the stimulation of extracellular signals [6, 15]. In the present study, we detected a slight enhancement in the mRNA levels of genes associated with the Ca^2+^ signaling pathway in response to treatment with Zn^2+^, (Table 3), thereby indicating that *plc-e* might play a role in the release of Ca^2+^ from intracellular Ca^2+^ pools. Treatment with LaCl_3_ and deletion of *crz1* to block cytosolic Ca^2+^ signaling indicated that calcium signal transduction is partially involved in the early phase of Zn^2+^ induction, thereby implying the involvement of other pathways in the Zn^2+^-induced excessive production of cellulase (Additional file 3: Fig. S3, Additional file 4: Fig. S4), which warrants further investigation.

ZafA, an important transcription factor, plays a key role in maintaining Zn^2+^ homeostasis, which has been shown to be associated with gliotoxin biosynthesis in *A. fumigatus* [30]. In the present study, RNA-seq analysis revealed that the expression of one zinc-finger protein was significantly downregulated in response to treatment with Zn^2+^ (Additional file 5: Table S1). On the basis of an NCBI blastx search, we established that this protein is homologous to *zap1* in *S. cerevisiae* [39] and *zafA* in *A. fumigatus* [33], and we accordingly designated the protein *zafA*. Phylogenetic tree analysis revealed that *zafA* is expressed in a range of fungi, particularly species in the genus *Trichoderma* (Fig. 6b). With respect to fungi, transcription factors have been studied primarily in pathogenic species such as *A. fumigatus* [47] and *C. albicans* [48]. To date, however, the role of *zafA* in Zn^2+^ homeostasis has yet to be reported in cellulase-producing strains. In the present study, we demonstrated that following the deletion of *zafA*, the efficacy of Zn^2+^ in promoting cellulase production and the expression of cellulase-related genes was markedly attenuated, and in some case was completely inhibited (Fig. 7). Collectively, these observations would thus tend to indicate that *zafA* plays a prominent role in *T. reesei* when subjected to Zn^2+^ stress.

Exposure of *T. reesei* Rut-C30 to sufficiently high concentrations of Zn^2+^ was found induce stress-like responses in the fungi, and consequently, we examined the expression levels of two major antioxidant enzymes (*sod1* and *cat1* [46]) in response to 3 mM Zn^2+^ treatment based on RT-qPCR. We accordingly found that the mRNA levels of *sod1* were markedly enhanced at 36 h and 60 h, which was not substantially altered by the addition of Zn^2+^ at 48 h (Additional file 9: Fig. S6). Contrastingly, the levels of *cat1* were observed to undergo a gradual, albeit marked, decline with time (Additional file 9: Fig. S6). These findings thus provide evidence indicating that cells were subjected to oxidative stress in response to treatment with Zn^2+^. Similar findings have been reported for *S. cerevisiae*, in which elevated levels of intracellular ROS have been detected in zinc-sensitive mutants exposed to high Zn^2+^ stress [27]. In our previous study, we established that exposure to 70 mM Sr^2+^ promoted a ROS burst and associated increases in the expression of *sod1* and *cat1* in *T. reesei* [46]. Similarly, Chen et al. [49] demonstrated that the mRNA levels of antioxidant enzyme genes in *P. brevicompactum* were markedly upregulated in response to Ca^2+^. Treatment with metal ions exposes *T. reesei* Rut-C30 to stress, in response to which there is an upregulated expression of antioxidant genes to cope with the danger, and in our aforementioned previous study, we demonstrated that a ROS scavenger can alleviate intracellular ROS promoted by exposure to Sr^2+^ to enhance cellulase production [46]. In further studies, we accordingly intend to examine the effects of simultaneous treatment with Zn^2+^ and ROS scavengers on cellulase hyperproduction in RUT-C30.

In the presence of Zn^2+^, we detected a significant enhancement in the transcription levels of *xyr1* and *ace3* (Fig. 3f, g and Table 2), which are vital positive transcription activators that regulate cellulase gene expression in *T. reesei* [9, 12]. It is reasonable to speculate that *zafA* enhances the transcription of cellulase genes or associated activators. However, further studies will be necessary to establish the mechanisms underlying *zafA* recognition of the promoter sequences of these genes. Furthermore, we also detected the prominently enhanced expression of *vib1* (Table 2), the deletion of which had the effect of reducing the expression of almost all cellulase and hemicellulase genes, vital sugar transporter genes, and the primary transcriptional activators *xyr1* and *ace3* in *T. reesei* [49]. In this regard, it has been found that in *Neurospora crassa*, Vib1 modulates cellulase synthesis by regulating the expression of the essential cellulase regulator CLR2 [50]. In the present study, we found the upregulation of *vib1* to be consistent with the activation of *xyr1* and *ace3* in response to Zn^2+^. Furthermore, Zn^2+^ stimulation was found to be associated with the differential expression of 17 GPCRs and 44 transporter genes, which accordingly warrant further investigation with respect to their role in altered membrane signaling.

## Conclusions

**Fig. 8:**
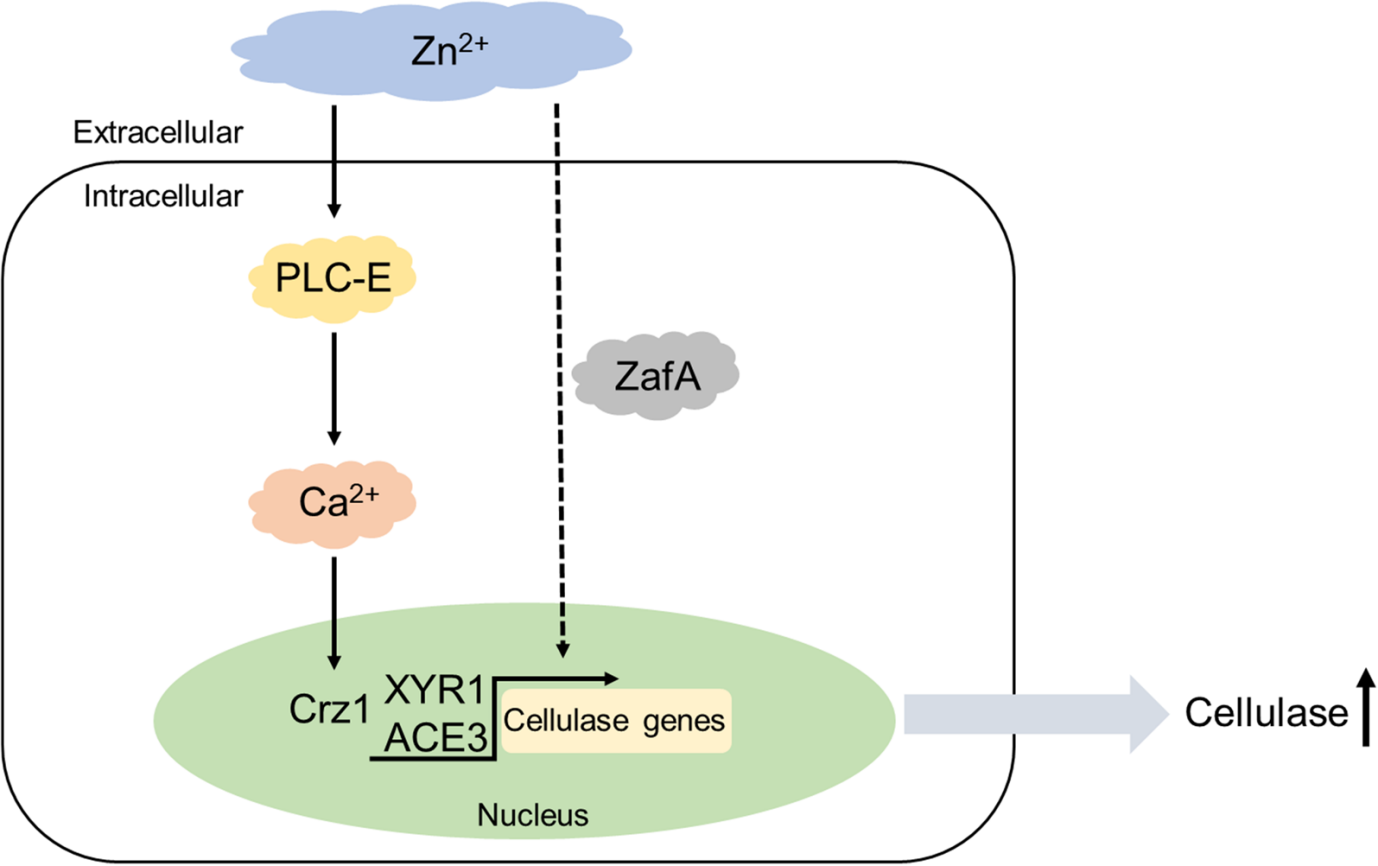
Putative mechanism of Zn^2+^ induction of cellulase production in *Trichoderma reesei*. Supplementation of medium with 3 mM Zn^2+^ enhanced cellulase production and the expression of *plc-e* and calcium signaling genes. Disruption of *plc-e*, treatment with LaCl_3_ (a plasma membrane Ca^2+^ channel blocker), and the deletion of *crz1* (calcineurin-responsive zinc-finger transcription factor 1) revealed that calcium signaling is partially involved in the induction process. Moreover, there were marked reduction in mRNA levels of the zinc-responsive transcription factor *zafA* in response to Zn^2+^ stimulation. Deletion of *zafA* indicated that this transcription factor plays a prominent role in mediating Zn^2+^-induced excessive production of cellulase. The solid arrows indicate data supported by our results, and the dashed arrows indicate undefined regulation

In this study, we discovered that supplementation of medium with 3 mM Zn^2+^ markedly inhibited the hyphal growth of *T. reesei* Rut-C30, whereas this treatment promoted a substantial enhancement in cellulase and xylanase production. Furthermore, we detected a significant upregulation of the transcription of major cellulase and xylanase genes, as well as that of two vital transcriptional activator genes (*ace3* and *xyr1*). Transcriptional analysis revealed that the expression of *plc-e* was similarly significantly upregulated. We established that Zn^2+^ modulates the expression of cellulase genes, partly via the *plc-e* gene and Ca^2+^ signal transduction. In addition, we identified a transcription factor, *zafA*, associated with Zn^2+^ homeostasis, which was found to play a prominent role in Zn^2+^-induced excessive production of cellulase. On the basis of our findings, we thus elucidated a putative mechanism whereby Zn^2+^ regulates cellulase production in *T. reesei* (Fig. 8). Our identification of a novel inducer that promotes cellulase synthesis and the mechanisms underlying induction provide a valuable basis for further research on Zn^2+^ signal transduction in *T. reesei*.

## Methods

### Strains and growth conditions

*Escherichia coli* DH5α cells were used as the host for plasmid amplification. Fungal transformation was based on infection using the GV3101 strain of *Agrobacterium tumefaciens* [51]. *T. reesei* Rut-C30 (ATCC 56765) [52] was used as the host strain for genetic transformation. Luria broth (LB) was used to culture *E. coli* and *A. tumefaciens*, and MA medium [12] containing 2% (w/v) glucose and MM [46] containing 1% (w/v) Avicel or 2% (w/v) glucose were used for general fungal culture. All strains of *T. reesei* were maintained on potato dextrose agar (PDA) plates in the dark at 28 °C. Fresh conidia were collected using glycerin for subsequent analyses. MM, which was used to assess the effect of Zn^2+^ on cellulase production and hyphal growth, contained (g/L) (NH_4_)_2_SO_4_ (5), urea (0.3), KH_2_PO_4_ (15), CaCl_2_ (0.6), MgSO_4_ (0.6), MnSO_4_·H_2_O (0.0016), FeSO_4_·7H_2_O (0.005), and CoCl_2_·6H_2_O (0.002), pH 5.5, with 1% (w/v) Avicel or 2% (w/v) glucose as the sole carbon source. Conidia (2 × 10^6^) were grown for 36 h at 28 °C in 100 mL of MA medium supplemented with 2% (w/v) glucose as the sole carbon source (220 rpm). Equal amounts of mycelia (approximately 0.1 g) were collected and washed thoroughly using 20 mL of fresh MM lacking a carbon source, and then transferred to 50 mL of fresh MM containing 1% (w/v) Avicel, with the addition of 0–5 mM ZnCl_2_. After 2–4 days of culture, samples were collected for enzymatic activity and protein concentration determinations. Mycelia induced for 36, 48, or 60 h were collected and maintained frozen at − 80 °C for subsequent RNA isolation and RT‑qPCR analyses.

### Construction of *T. reesei* Δ*plc-e,* Δ*zafA*, and Δ*crz1* mutants

In this study, RUT-C30 was used as the parent strain for gene knockout. A pEASY^®^-Uni Seamless Cloning and Assembly Kit (TransGen, Shanghai, China) was used to construct deletion cassettes for deletion of the *plc-e*, *zafA*, and *crz1* genes. The primers used for plasmid construction and diagnostic PCR are listed in Additional file 10: Table S4. Specifically, 722-bp upstream and 757-bp downstream fragments of *plc-e* were generated from the RUT-C30 genome using KOD-Plus-Neo (TOYOBO, Osaka, Japan). Initially, the upstream fragment obtained by PCR was ligated into *Pac*I- and *Xba*I-linearized LML2.1 [53] to generate pF*plc-e*. Subsequently, the downstream fragment was inserted into the *Swa*I sites of pF*plc-e* to the generate deletion binary pD*plc-e* (Additional file 11: Fig. S7). Similarly, the deletion cassettes of *zafA* and *crz1* were amplified and inserted into LML2.1 to generate the deletion vectors pD*zafA* and pD*crz1*, respectively. Deletion cassettes were used to transform *T. reesei* RUT-C30 to facilitate the knockout of *plc-e* (Δ*plc-e* mutant), *zafA* (Δ*zafA* mutant), and *crz1* (Δ*crz1* mutant) using *Agrobacterium*-mediated transformation [54]. The xylose-induced Cre recombinase system was used to self-excise the hygromycin-resistant cassette [55]. Primers XX-CF, XX-CR, XX-OF, and XX-OR (XX represents the gene name) were used in diagnostic PCR to verify the putative gene disruption mutants generated by double crossover.

### Fungal growth and cellulase, xylanase, protein, and biomass production

For fungal hyphal growth assays, glycerin was used to collect fresh conidia, which were diluted to 2.5 × 10^6^ mL^−1^ in sterile water. An equal volume of diluted conidia (2 μL) was inoculated onto the center of MM plates containing 2% (w/v) glucose and incubated for 4 days at 28 °C. Cellulase, xylanase, protein, and biomass production assays were performed as previously described [46, 56].

### RNA isolation and quantitative real-time reverse transcription polymerase chain reaction (RT-qPCR)

The mRNA levels of specific gene mRNAs were assessed using RT-qPCR, as described by Cai et al. [52]. Briefly, total RNA from 100 mg of *T. reesei* mycelia was extracted using a FastRNA Pro Red Kit (MPbio, Irvine, CA, USA) according to the manufacturer’s instructions. For reverse transcription, we used *TransScript*^®^ All-in-One First-Strand cDNA Synthesis SuperMix for qPCR (One-Step gDNA Removal) (TransGen Biotech, Beijing, China) was used to reverse-transcribe 800 ng of total RNA to produce cDNA. RT-qPCR was performed using PerfectStart™ Green qPCR SuperMix (TransGen Biotech) with 1 μL of cDNA and 200 nM of the forward and reverse primers in a final volume of 20 μL. The sequences of the primers used in the RT-qPCR analysis are shown in Additional file 10: Table S4. For gene transcription analysis, the *sar1* gene was used as the reliable reference in SYBR Green assays as previously described [57]. For RT-qPCR analysis, thermocycling was performed using an ABI StepOne thermocycler (Applied Biosystems, Foster City, CA, USA).

### Chemical reagent treatments

For an assessment of Zn^2+^ stress, we added different concentrations of ZnCl_2_ to fungal growth medium. For plate growth studies, *T. reesei* was cultured on solid MM for 4 days. Different concentrations of ZnCl_2_ were added immediately after the mycelia had been transferred to the medium. For enzyme production analysis, conidia were germinated in MA medium to yield mycelia, which were subsequently transferred to MM supplemented with different concentrations of ZnCl_2_. To examine the roles of cytosolic Ca^2+^ in response to Zn^2+^ stress, mycelia were also treated with LaCl_3_ (a plasma membrane Ca^2+^ channel blocker), which was used at a final concentration of 5 mM after 1 day in *T. reesei* culture.

### Whole-transcriptome shotgun sequencing (RNA‑seq) analysis

*T. reesei* RUT-C30 mycelia, treated with 0 or 3 mM Zn^2+^ in liquid MM containing 1% (w/v) Avicel as the sole carbon source, were harvested after 48 h for RNA-seq. For sequencing, all treated and untreated strains were sent to Shanghai Majorbio Bio-pharm Technology Co., Ltd. (Shanghai, China) in triplicate. The latest reference genome of *T. reesei* (https://www.ncbi.nlm.nih.gov/assembly/GCA_002006585.1) was used in this study for bioinformatic analysis. The whole-transcriptome data have been submitted to the NCBI SRA website (https://www.ncbi.nlm.nih.gov/sra/PRJNA923496) with the Accession Number PRJNA923496.

### Statistical analysis

In this study, at least three independent experiments were performed to obtain reliable data with identical or similar results. The standard deviations (SDs) from the mean of triplicate determinations are indicated by error values. Student’s *t* test or Duncan’s multiple-range test was used for bicomponent or multiple comparisons, respectively. Within each set of experiments, *p* < 0.05 was considered to indicate significant differences between the data.

## Supplementary Information

**Additional file 1: Figure S1. **The heat map showed the correlation between the biological replicates of each sample in RNA‑seq analysis. The value in the square is the correlation coefficient between the two samples. The larger the value, the greater the correlation between the two samples and the closer they are. These results mean that the RNA‑seq data is very reliable.

**Additional file 2: Figure S2.** Effect of 3 mM Zn^2+^ on calcium signal transduction related gene transcription levels in RUT-C30 strain. Transcriptional levels of *cam* (**a**), *cna1* (**b**), and *crz1* (**c**) of the RUT-C30 strain were detected after culturing in liquid MM for 36, 48 or 60 h containing 0 or 3 mM Zn^2+^ with 1% (w/v) Avicel as the sole carbon source. The final values are presented as the mean±standard deviation (SD) of three independent experimental results. Asterisks indicate significant differences compared to the control (*P <0.05, according to Student’s* t*-test).

**Additional file 3: Figure S3.** Effect of LaCl_3_ on cellulase production after Zn^2+^ treatment. *p*NPCase (**a**) and CMCase (**b**) activity were measured in the RUT-C30 strain after exposed to Zn^2+^ or LaCl_3_. The transcriptional levels of *cbh1* (**c**) and *egl1* (**d**) were detected after culturing the RUT-C30 strain in medium supplemented with 0 or 3 mM Zn^2+^ and with (+) or without (−) 5 mM LaCl_3_. The final values are presented as the mean±standard deviation (SD) of three independent experimental results. Asterisks indicate significant differences compared to the control (*P <0.05, according to Student’s* t*-test).

**Additional file 4: Figure S4.** Effect of* crz1* on cellulase production after Zn^2+^ addition.* p*NPCase activity (**a**), CMCase activity (**b**) of RUT-C30 and Δ*crz1* cultured in liquid MM for 2, 3, or 4 days with or without 3 mM Zn^2+^ and 1% (w/v) Avicel as the sole carbon source, respectively. The expression levels of *cbh1* (**c**) and *egl1 *(**d**) were also determined. The final values are presented as the mean±standard deviation (SD) of three independent experimental results. Asterisks indicate significant differences compared to the control (*P <0.05, according to Student’s *t*-test).

**Additional file 5: Table S1.** The mRNA level of* zafA *(M419DRAFT_96242) was detected by RNA‑seq analysis.

**Additional file 6: Figure S5.** Effect of 3 mM Zn^2+^ on *zafA *transcription level in RUT-C30 strain. Transcriptional level of *zafA* of the RUT-C30 strain were detected after culturing in liquid MM for 36, 48 or 60 h containing 0 or 3 mM Zn^2+^ with 1% (w/v) Avicel as the sole carbon source. The final values are presented as the mean±standard deviation (SD) of three independent experimental results. Asterisks indicate significant differences compared to the control (*P <0.05, according to Student’s* t*-test).

**Additional file 7: Table S2. **The changes of 58 GPCR genes [1] in response to Zn^2+^ stimulus, NS represented not significant, *p* adjust>0.05

**Additional file 8: Table S3. **The changes of 152 transporter genes in response to Zn^2+^ stimulus, NS represented not significant, *p* adjust>0.05

**Additional file 9: Figure S6.** Effect of 3 mM Zn^2+^ on two major antioxidant enzyme gene transcription levels in RUT-C30 strain. Transcriptional levels of* sod1* (**a**) and *cat1* (**b**) of the RUT-C30 strain were determined after culturing in liquid MM for 36, 48 or 60 h containing 0 or 3 mM Zn^2+^ with 1% (w/v) Avicel as the sole carbon source. The final values are presented as the mean±standard deviation (SD) of three independent experimental results. Asterisks indicate significant differences compared to the control (*P <0.05, according to Student’s* t*-test).

**Additional file 10: Table S4. **Primers used in this study.

**Additional file 11: Figure S7.** Construction and verification of *T. reesei* deletion mutant, which were performed as described in our previous study [1]. **a**: The skeleton schematic diagram to delete *plc-e* in the parent strain RUT-C30. **b**: The skeleton schematic diagram to delete* zafA* in the parent strain RUT-C30. **c**: The skeleton schematic diagram to delete* crz1* in the parent strain RUT-C30. **d**: Validated electrophoretic diagram to verify the knockout of *plc-e*, *zafA*, and *crz1* in the parent strain RUT-C30, respectively; M: marker.

## Data Availability

All data generated or analyzed in this study are available and included in this published article and its Additional information files.

## Abbreviations

CMCase: *endo*-β-Glucanase activity
*crz1*: Calcineurin-responsive zinc-finger transcription factor 1
DEG: Differentially expressed gene
FPase: Total extracellular cellulase activity
GPCR: G-protein-coupled receptor
MM: Minimal medium
PDA: Potato dextrose agar
*p*NPCase: Exo-β-glucanase activity
RNA‑seq: Whole-transcriptome shotgun sequencing
ROS: Reactive oxygen species
RT-qPCR: Quantitative real-time reverse transcription polymerase chain reaction
TF: Transcription factor
