# Supplementary material for: Mechanism of Zn2+ regulation of cellulase production in Trichoderma reesei Rut-C30
Source: Biotechnol Biofuels Bioprod. 2023 Apr 28;16:73. doi: 10.1186/s13068-023-02323-1 (PMC10148476; doi:10.1186/s13068-023-02323-1)
Supplement: Supplementary file 1 — Additional file 1: Figure S1. The heat map showed the correlation between the biological replicates of each sample in RNA‑seq analysis. The value in the square is the correlation coefficient between the two samples. The larger the value, the greater the correlation between the two samples and the closer they are. These results mean that the RNA‑seq data is very reliable. [file 13068_2023_2323_MOESM1_ESM.docx]

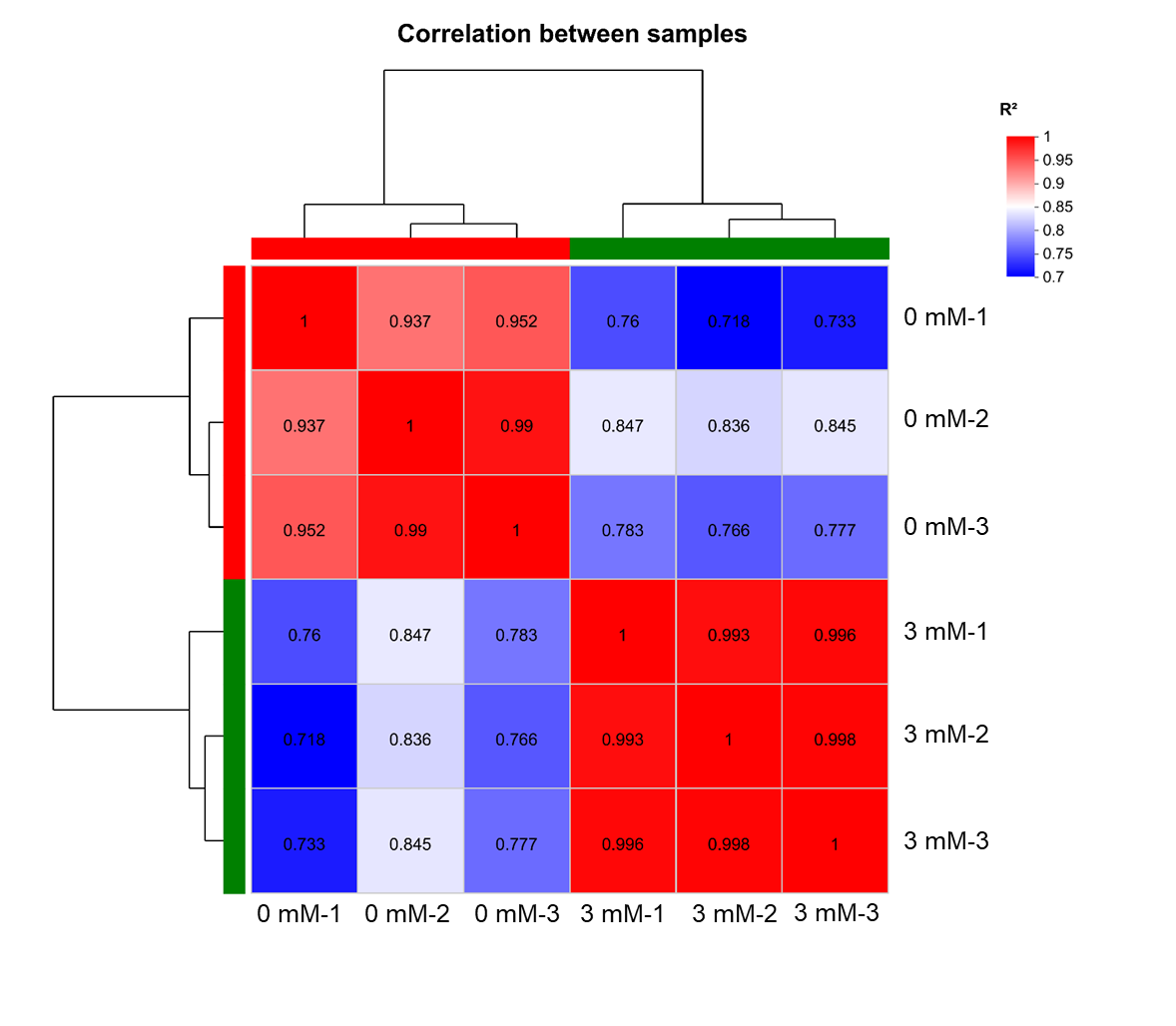


**Fig. S1** The heat map showed the correlation between the biological replicates of each sample in RNA‑seq analysis. The value in the square is the correlation coefficient between the two samples. The larger the value, the greater the correlation between the two samples and the closer they are. These results mean that the RNA‑seq data is very reliable.
