# Supplementary material for: Mechanism of Zn2+ regulation of cellulase production in Trichoderma reesei Rut-C30
Source: Biotechnol Biofuels Bioprod. 2023 Apr 28;16:73. doi: 10.1186/s13068-023-02323-1 (PMC10148476; doi:10.1186/s13068-023-02323-1)
Supplement: Supplementary file 3 — Additional file 3: Figure S3. Effect of LaCl3 on cellulase production after Zn2+ treatment. pNPCase (a) and CMCase (b) activity were measured in the RUT-C30 strain after exposed to Zn2+ or LaCl3. The transcriptional levels of cbh1 (c) and egl1 (d) were detected after culturing the RUT-C30 strain in medium supplemented with 0 or 3 mM Zn2+ and with (+) or without (−) 5 mM LaCl3. The final values are presented as the mean±standard deviation (SD) of three independent experimental results. Asterisks indicate significant differences compared to the control (*P <0.05, according to Student’s t-test). [file 13068_2023_2323_MOESM3_ESM.docx]

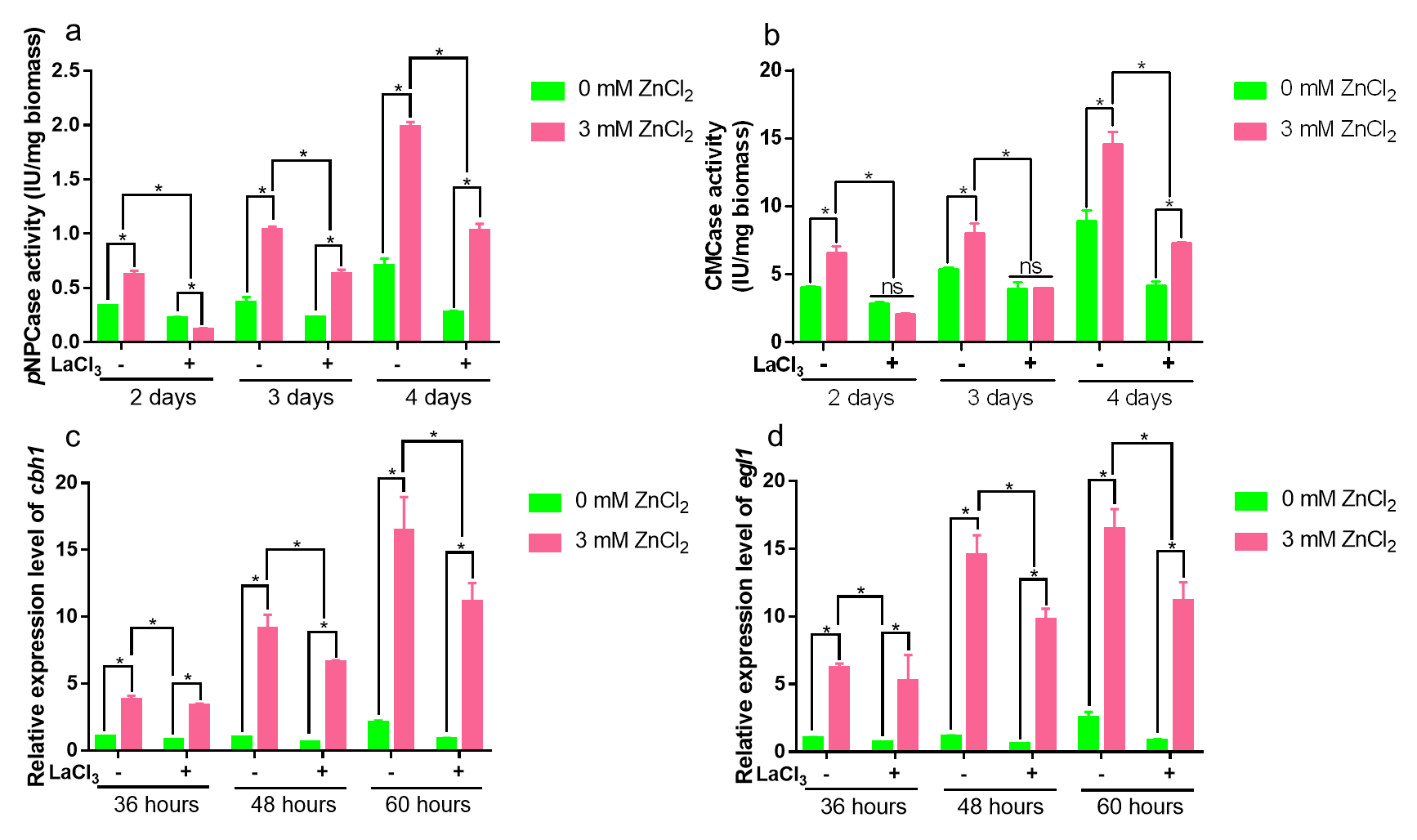


**Fig. S3** Effect of LaCl_3_ on cellulase production after Zn^2+^ treatment. *p*NPCase (**a**) and CMCase (**b**) activity were measured in the RUT-C30 strain after exposed to Zn^2+^ or LaCl_3_. The transcriptional levels of *cbh1* (**c**) and *egl1* (**d**) were detected after culturing the RUT-C30 strain in medium supplemented with 0 or 3 mM Zn^2+^ and with (+) or without (−) 5 mM LaCl_3_. The final values are presented as the mean±standard deviation (SD) of three independent experimental results. Asterisks indicate significant differences compared to the control (**P* <0.05, according to Student’s *t*-test).
