# Supplementary material for: Mechanism of Zn2+ regulation of cellulase production in Trichoderma reesei Rut-C30
Source: Biotechnol Biofuels Bioprod. 2023 Apr 28;16:73. doi: 10.1186/s13068-023-02323-1 (PMC10148476; doi:10.1186/s13068-023-02323-1)
Supplement: Supplementary file 4 — Additional file 4: Figure S4. Effect of crz1 on cellulase production after Zn2+ addition. pNPCase activity (a), CMCase activity (b) of RUT-C30 and Δcrz1 cultured in liquid MM for 2, 3, or 4 days with or without 3 mM Zn2+ and 1% (w/v) Avicel as the sole carbon source, respectively. The expression levels of cbh1 (c) and egl1 (d) were also determined. The final values are presented as the mean±standard deviation (SD) of three independent experimental results. Asterisks indicate significant differences compared to the control (*P <0.05, according to Student’s t-test). [file 13068_2023_2323_MOESM4_ESM.docx]

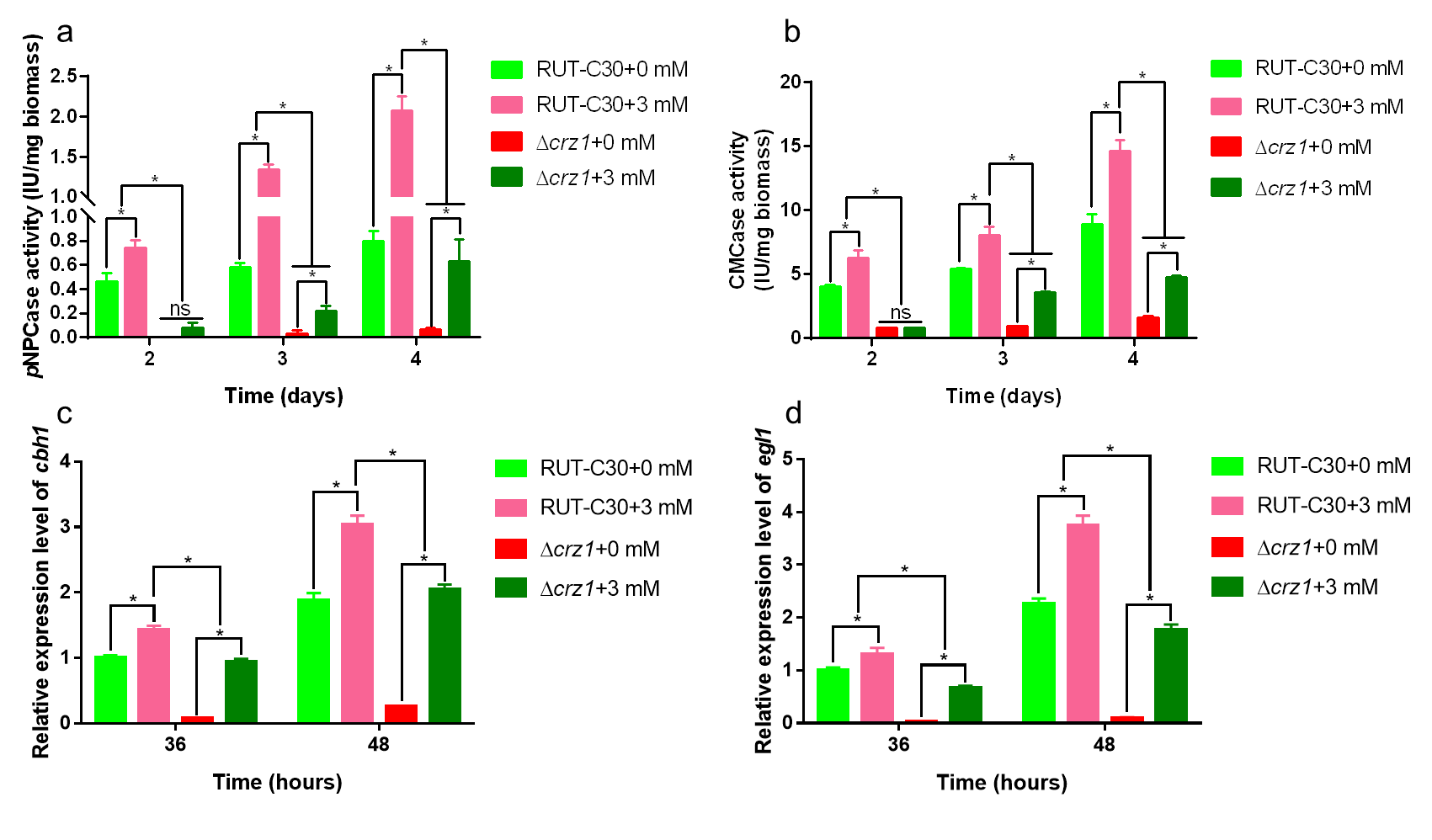


**Fig. S4** Effect of *crz1* on cellulase production after Zn^2+^ addition. *p*NPCase activity (**a**), CMCase activity (**b**) of RUT-C30 and Δ*crz1* cultured in liquid MM for 2, 3, or 4 days with or without 3 mM Zn^2+^ and 1% (w/v) Avicel as the sole carbon source, respectively. The expression levels of *cbh1* (**c**) and *egl1* (**d**) were also determined. The final values are presented as the mean±standard deviation (SD) of three independent experimental results. Asterisks indicate significant differences compared to the control (**P* <0.05, according to Student’s *t*-test).
