# Supplementary material for: Mechanism of Zn2+ regulation of cellulase production in Trichoderma reesei Rut-C30
Source: Biotechnol Biofuels Bioprod. 2023 Apr 28;16:73. doi: 10.1186/s13068-023-02323-1 (PMC10148476; doi:10.1186/s13068-023-02323-1)
Supplement: Supplementary file 5 — Additional file 5: Table S1. The mRNA level of zafA (M419DRAFT_96242) was detected by RNA‑seq analysis. [file 13068_2023_2323_MOESM5_ESM.docx]

**Table S1** The mRNA level of *zafA* (M419DRAFT_96242) was detected by RNA‑seq analysis.

| **Gene ID** | **Log_2_fc** | **Up/Down** | ***p* adjust** |
| --- | --- | --- | --- |
| 96242 | -0.693075386 | Slightly Down | 1.14933E-07 |
