# Supplementary material for: Mechanism of Zn2+ regulation of cellulase production in Trichoderma reesei Rut-C30
Source: Biotechnol Biofuels Bioprod. 2023 Apr 28;16:73. doi: 10.1186/s13068-023-02323-1 (PMC10148476; doi:10.1186/s13068-023-02323-1)
Supplement: Supplementary file 6 — Additional file 6: Figure S5. Effect of 3 mM Zn2+ on zafA transcription level in RUT-C30 strain. Transcriptional level of zafA of the RUT-C30 strain were detected after culturing in liquid MM for 36, 48 or 60 h containing 0 or 3 mM Zn2+ with 1% (w/v) Avicel as the sole carbon source. The final values are presented as the mean±standard deviation (SD) of three independent experimental results. Asterisks indicate significant differences compared to the control (*P <0.05, according to Student’s t-test). [file 13068_2023_2323_MOESM6_ESM.docx]

**Fig. S5** Effect of 3 mM Zn^2+^ on *zafA* transcription level in RUT-C30 strain. Transcriptional level of *zafA* of the RUT-C30 strain were detected after culturing in liquid MM for 36, 48 or 60 h containing 0 or 3 mM Zn^2+^ with 1% (w/v) Avicel as the sole carbon source. The final values are presented as the mean±standard deviation (SD) of three independent experimental results. Asterisks indicate significant differences compared to the control (**P* <0.05, according to Student’s *t*-test).
