# Supplementary material for: Mechanism of Zn2+ regulation of cellulase production in Trichoderma reesei Rut-C30
Source: Biotechnol Biofuels Bioprod. 2023 Apr 28;16:73. doi: 10.1186/s13068-023-02323-1 (PMC10148476; doi:10.1186/s13068-023-02323-1)
Supplement: Supplementary file 7 — Additional file 7: Table S2. The changes of 58 GPCR genes [1] in response to Zn2+ stimulus, NS represented not significant, p adjust>0.05 [file 13068_2023_2323_MOESM7_ESM.docx]

**Table S2** The changes of 58 GPCR genes [1] in response to Zn^2+^ stimulus, NS represented not significant, *p* adjust>0.05

| **Gene ID** | **GPCR class** | **Log_2_fc** | ***p* adjust** | **Up/Down** |
| --- | --- | --- | --- | --- |
| 42876 | I (pheromone receptors) | -0.241625667 | 0.63873789 | NS |
| 70769 | II (pheromone receptors) | -1.184466112 | 0.584925295 | NS |
| 75467 | III (related to A. nidulans GprC, GprD, and GprE) | -1.014837081 | 0.000212703 | Down |
| 101496 | IV (nitrogen sensors) | 0.01171816 | 0.968382285 | NS |
| 82587 | IV (nitrogen sensors) | 0.626977897 | 0.000924154 | Up |
| 138986 | V (cAMP receptor-like) | -2.509247911 | 7.30872E-44 | Down |
| 86058 | V (cAMP receptor-like) | × | × | × |
| 6450 | V (cAMP receptor-like) | × | × | × |
| 73489 | V (cAMP receptor-like) | 0 | 1 | NS |
| 82265 | VI (GPCRs containing RGS domain) | 0.714458013 | 0.015821027 | Up |
| 142712 | VI (GPCRs containing RGS domain) | 1.972088203 | 1.04189E-48 | Up |
| 134387 | VI (GPCRs containing RGS domain) | 1.357462681 | 2.81168E-06 | Up |
| 69999 | VII (related to rat growth hormone releasing factor) | 1.487970166 | 7.70519E-25 | Up |
| 139847 | VIII (related to human steroid receptor mPR) | -0.328777379 | 0.705619768 | NS |
| 88513 | VIII (related to human steroid receptor mPR) | 0.186080109 | 0.495607158 | NS |
| 39456 | VIII (related to human steroid receptor mPR) | -1.268765481 | 0.195283314 | NS |
| 104559 | VIII (related to human steroid receptor mPR) | 0.821193816 | 0.017251418 | Up |
| 72746 | VIII (related to human steroid receptor mPR) | 0.233235028 | 0.322393832 | NS |
| 134859 | X (similar to PTM1) | 0.233977788 | 0.107922454 | NS |
| 81195 | XI (similar to GPCR89) | 1.227649389 | 6.50373E-12 | Up |
| 32680 | XII (family C-like GPCRs) | -0.07006486 | 0.877299167 | NS |
| 106845 | XIII (related to GPR11 of P. sojae) | -0.066075597 | 0.742696075 | NS |
| 11680 | XIII (related to GPR11 of P. sojae) | 0.533049158 | 0.100488507 | NS |
| 12444 | PTH11-like GPCRs | -0.688401662 | 1 | NS |
| 85523 |  | -0.121200755 | 0.62689754 | NS |
| 107912 |  | -0.987650975 | 0.000315889 | Up |
| 12050 |  | 3.639965051 | 0.273957488 | NS |
| 75975 |  | 1.043463586 | 0.002347585 | Up |
| 7706 |  | 1.095665044 | 0.422052798 | NS |
| 95046 |  | 1.23445408 | 1.97224E-11 | Up |
| 69071 |  | 0 | 1 | NS |
| 46962 |  | -0.098217801 | 0.876435107 | NS |
| 76201 |  | -1.015229836 | 0.000160156 | Down |
| 134160 |  | -0.077530271 | 0.909265526 | NS |
| 87338 |  | 1.235669416 | 5.69075E-30 | Up |
| 129578 |  | 1.232383969 | 0.008633713 | Up |
| 101894 |  | 0.154388384 | 0.873425585 | NS |
| 64485 |  | 0.150059139 | 0.825131322 | NS |
| 133932 |  | 1.233402375 | 2.43387E-06 | Up |
| 24205 |  | 1.513154271 | 1.23915E-08 | Up |
| 135135 |  | 0 | 1 | NS |
| 79810 |  | 0.19480963 | 0.313651588 | NS |
| 90931 |  | 0.273381799 | 1 | NS |
| 12750 |  | 1.28373236 | 9.5412E-06 | Up |
| 133409 |  | -0.422276463 | 0.67209247 | NS |
| 6709 |  | 2.422889218 | 0.009684521 | Up |
| 48593 |  | 2.467571251 | 0.370519548 | NS |
| 104737 |  | 0.946771226 | 1.10378E-15 | Up |
| 142488 |  | × | × | × |
| 66673^a^ |  | 3.351122767 | 0.201232775 | NS |
| 136598 |  | 0.032794978 | 0.909123897 | NS |
| 76433 |  | 0 | 1 | NS |
| 94668 |  | 0.874472375 | 2.55157E-10 | Up |
| 83299 |  | -1.094247172 | 0.581433697 | NS |
| 95052 |  | 0.980772457 | 7.79601E-06 | Up |
| 78204 |  | 1.437034701 | 0.003291628 | Up |
| 5756 |  | 0.528938343 | 0.050375641 | NS |
| 104224 |  | 0.349771071 | 0.321733946 | NS |

× represents not detected; a represents the gene ID come from *T. reesei* QM6a.

**References**

1. Gruber S, Omann M, Zeilinger S. Comparative analysis of the repertoire of G protein-coupled receptors of three species of the fungal genus *Trichoderma*. BMC Microbiol. 2013;13:108.
