# Supplementary material for: Mechanism of Zn2+ regulation of cellulase production in Trichoderma reesei Rut-C30
Source: Biotechnol Biofuels Bioprod. 2023 Apr 28;16:73. doi: 10.1186/s13068-023-02323-1 (PMC10148476; doi:10.1186/s13068-023-02323-1)
Supplement: Supplementary file 8 — Additional file 8: Table S3. The changes of 152 transporter genes in response to Zn2+ stimulus, NS represented not significant, p adjust>0.05 [file 13068_2023_2323_MOESM8_ESM.docx]

**Table S3** The changes of 152 transporter genes in response to Zn^2+^ stimulus, NS represented not significant, *p* adjust>0.05

| **Gene ID** | **Description** | **Log_2_fc** | ***p* adjust** | **Up/Down** |
| --- | --- | --- | --- | --- |
| 75479 | MFS permease | 0.466353429 | 0.010920334 | Up |
| 37067 | AAA ATPase | 0.035981812 | 0.854946489 | NS |
| 94212 | Synaptic vesicle transporter | -0.359989583 | 0.27554728 | NS |
| 98410 | MFS permease | -0.150069413 | 0.945017569 | NS |
| 93825 | Ammonia permease | 1.194557681 | 3.79127E-12 | Up |
| 97114 | MitochoNSrial intermediate peptidase | 0.531407663 | 0.000177721 | Up |
| 88258 | Nuclear pore complex, Nup155 | -0.194000947 | 0.200442151 | NS |
| 126296 | MFS permease | 2.468049524 | 0.000291218 | Up |
| 36248 | Nuclear GTPase 1 | 0.756276441 | 0.000490659 | Up |
| 113484 | Amino acid transporters | 0.407606747 | 0.005605997 | Up |
| 98774 | MFS permease | -2.572146823 | 0.409738482 | NS |
| 89987 | Amino acid transporter | 0.14268605 | 0.647643744 | NS |
| 23525 | Siderophore transporter | 1.047549869 | 4.63813E-05 | Up |
| 70134 | Fatty acid transporter | 0.315362559 | 0.281566527 | NS |
| 99152 | Golgi transport complex subunit | 0.462816145 | 0.003352994 | Up |
| 90646 | MFS permease | 1.109942424 | 1.12427E-14 | Up |
| 73724 | AAA ATPase | -0.509925129 | 7.03093E-06 | Down |
| 33711 | Voltage-gated shaker-like K+ channel | -0.073337657 | 0.842494847 | NS |
| 131359 | Tetracycline resistance protein TetB | -0.237831245 | 0.445126861 | NS |
| 73108 | MFS permease | -1.246792319 | 2.70126E-09 | Down |
| 132197 | Tetracycline resistance protein | -1.201714167 | 0.009787186 | Down |
| 67631 | MFS permease | -0.093903896 | 0.87444728 | NS |
| 133137 | Nuclear migration protein | 0.228103873 | 0.247283871 | NS |
| 83372 | Cation transporting ATPase | -0.345727964 | 0.492224704 | NS |
| 94554 | Oligopeptide transporter | -2.673013201 | 7.17743E-13 | Down |
| 130751 | MFS permease | 1.297166075 | 2.49812E-13 | Up |
| 128469 | Diamine acetyltransferase | -0.47379971 | 0.840021169 | NS |
| 69350 | Amino acid transporter | -0.258346902 | 0.700132084 | NS |
| 52104 | Monocarboxylate transporter | -0.490882745 | 0.132689988 | NS |
| 134530 | Synaptic vesicle transporter SVOP | 0.896867311 | 0.000688732 | Up |
| 92898 | MFS permease | 0.752574714 | 0.686963753 | NS |
| 63513 | Ca-permeable channel | 0.060467079 | 0.872416378 | NS |
| 139450 | Amino acid permease Dip5 | -1.398037762 | 0.675362372 | NS |
| 108381 | Plasma membrane H^+^ ATPase | 0.397070332 | 0.089282527 | NS |
| 91594 | Maltose permease | 1.62302108 | 1.97884E-18 | Up |
| 134987 | Ca^2+^ transporter | -0.484832695 | 0.008852774 | Down |
| 70522 | Sugar transporter | 0.458454396 | 0.097988039 | NS |
| 7422 | Inorganic phosphate transporter | 1.231858827 | 3.52183E-16 | Up |
| 5560 | Vacuolar H^+^-ATPase V1 sector | 0.007173427 | 0.984314641 | NS |
| 143141 | Cytosine-purine permease | 1.453731577 | 1.41333E-09 | Up |
| 26932 | MFS permease | 0.111516006 | 0.831873354 | NS |
| 104814 | MFS permease | -0.534960181 | 0.01108847 | Down |
| 7909 | emp24/gp25L/p24 family of membrane trafficking proteins | 0.628738624 | 3.2295E-06 | Up |
| 33630 | Xylose transporter | 1.005660275 | 1.9323E-08 | Up |
| 142901 | Synaptic vesicle transporter SV2 | 1.745105925 | 9.59353E-27 | Up |
| 100875 | Amino acid transporters | 0.999891128 | 0.00634102 | Up |
| 90843 | Monocarboxylate transporter | -0.32241865 | 0.183728877 | NS |
| 86755 | Monocarboxylate transporter | -0.167599426 | 0.260481445 | NS |
| 11899 | MFS permease | -0.363925167 | 0.176402175 | NS |
| 86501 | Amino acid transporter | 0.422651674 | 0.057008183 | NS |
| 135121 | Monocarboxylate transporter | -1.286547382 | 0.000113331 | Down |
| 37832 | Monocarboxylate transporter | 1.971267902 | 4.39017E-07 | Up |
| 89596 | MFS permease | -0.933549638 | 0.007139684 | Down |
| 36469 | Monocarboxylate transporter | 0 | 1 | NS |
| 135838 | MFS permease | 0.40836995 | 0.382938451 | NS |
| 116127 | ABC transporter | -0.618630836 | 0.000200902 | Down |
| 91488 | Sugar transporter | 2.138781218 | 0.000415893 | Up |
| 97622 | Monocarboxylate transporter | -0.853082399 | 6.67912E-05 | Down |
| 105822 | ABC transporter-like protein | 0.256200816 | 0.156348598 | NS |
| 7811 | Sugar transporter | 1.007818859 | 1.3837E-10 | Up |
| 102186 | Sugar transporter | 0.109185513 | 0.544535656 | NS |
| 133770 | MFS permease | -0.237890405 | 0.336942829 | NS |
| 92240 | Aquaporin | 0.90930725 | 0.108257535 | NS |
| 26687 | Oligopeptide transporter | 0.349582629 | 0.22551451 | NS |
| 102778 | Amino acid transporters | 0.548631015 | 0.130828945 | NS |
| 84853 | MFS transport | 0.953984864 | 9.71572E-09 | Up |
| 84192 | Urea transporter | 0.178353302 | 0.479195641 | NS |
| 13268 | Synaptic vesicle transporter SVOP | 0.338086255 | 0.139774297 | NS |
| 135589 | Sugar transporter | 0.859363286 | 0.000330116 | Up |
| 109243 | MFS permease | 0.668839742 | 8.61442E-09 | Up |
| 142164 | H^+^/oligopeptide symporter | 1.468398409 | 1.18205E-19 | Up |
| 137407 | MitochoNSrial carrier protein | -0.708557344 | 7.98367E-07 | Down |
| 70312 | Sugar transporter | 0.638758659 | 0.005755911 | Up |
| 102402 | MFS permease | 1.442668651 | 2.09734E-05 | Up |
| 84399 | MFS permease | -0.024603675 | 0.943738797 | NS |
| 24422 | MFS permease | -0.244628695 | 0.319939416 | NS |
| 72297 | Urea transporter | 1.728126773 | 0.008874673 | Up |
| 138331 | Inner membrane transport protein yeiJ | 0.107870929 | 0.633393893 | NS |
| 97259 | MFS hexose transporter | 1.921681233 | 4.57428E-10 | Up |
| 77664 | Na^+^/K^+^ ATPase | -4.148895393 | 0.062856805 | NS |
| 137229 | MFS permease | 0.897777411 | 8.92269E-06 | Up |
| 69625 | MFS transporter | 0.184250894 | 0.710014855 | NS |
| 138519 | Sugar transporter | 1.210350096 | 9.83041E-10 | Up |
| 90786 | Amino acid transporters | -0.521416521 | 0.206009672 | NS |
| 89291 | Sugar transporter | 1.039080938 | 5.13843E-06 | Up |
| 102774 | MFS general substrate transporter | -0.018737576 | 0.947533072 | NS |
| 77618 | MFS permease | -0.451023718 | 0.207141747 | NS |
| 108217 | Uridine permease | -0.332730252 | 0.06749183 | NS |
| 101532 | Quinate permease | 0.974230115 | 0.000166276 | Up |
| 104585 | MFS permease | 0.368451926 | 0.085490104 | NS |
| 69901 | Sulphate anion transporter | -0.417673268 | 0.283341681 | NS |
| 127321 | Synaptic vesicle transporter SVOP | 0.055506027 | 0.779734089 | NS |
| 138789 | Amino acid transporters | 0.683935717 | 0.010419899 | Up |
| 136222 | MFS permease | 2.238103692 | 1.1835E-17 | Up |
| 79984 | Sugar transporter | 1.744111474 | 1.35081E-20 | Up |
| 131826 | Iron transporter | -2.286536926 | 9.12128E-51 | Down |
| 25063 | MFS permease | 0.110142319 | 0.539591518 | NS |
| 94416 | Lactate/pyruvate transporter | -1.10923271 | 3.49762E-11 | Down |
| 124396 | MFS permease (glucose permease HXT1) | -0.83591732 | 0.000968046 | Down |
| 8923 | Sugar transporter | 0.10167444 | 0.784206521 | NS |
| 11734 | Sugar transporter | 0.075653472 | 0.805054985 | NS |
| 6684 | Amino acid transporter PotE | 1.970371978 | 3.8546E-18 | Up |
| 126545 | Amino acid transporters | -0.054537356 | 0.905043462 | NS |
| 34838 | MitochoNSrial substrate carrier | -0.000386823 | 0.998326462 | NS |
| 133544 | Sugar transporter | 1.958971782 | 1.7736E-38 | Up |
| 12671 | Amino acid transporters | -0.95745221 | 0.001511158 | Down |
| 8867 | Amino acid transporters | 1.5470061 | 1.25562E-37 | Up |
| 26008 | MFS permease | 0.146826592 | 0.484586858 | NS |
| 91130 | MFS permease | 1.380189388 | 2.53426E-18 | Up |
| 69330 | MFS permease | 0.025595418 | 0.873009414 | NS |
| 98807 | Glycolipid transfer protein | -0.384112856 | 0.000993734 | Down |
| 93519 | MFS permease | -0.333665907 | 0.497817018 | NS |
| 68089 | Sugar transporter | -0.005643944 | 0.989321948 | NS |
| 104343 | Ammonia permease | 0.462171577 | 0.078042921 | NS |
| 94445 | MFS permease | -0.283494616 | 0.281068857 | NS |
| 106970 | C4-dicarboxylate transporter | 0.437105024 | 0.247268465 | NS |
| 73914 | ABC transporter | 0.181181454 | 0.276370871 | NS |
| 38765 | Sugar transporter | -0.177887901 | 0.67048865 | NS |
| 5378 | Synaptic vesicle transporter SVOP | 1.52991782 | 2.99805E-07 | Up |
| 75755 | Synaptic vesicle transporter SVOP | 1.338488922 | 7.33526E-17 | Up |
| 32968 | Amino acid transporters | 0.392559939 | 0.11861896 | NS |
| 7623 | Galactose permease | -2.206337411 | 1.10683E-20 | Down |
| 34769 | Molecular chaperone | -0.207835528 | 0.596146309 | NS |
| 80461 | ZIP zinc/iron transporter | -3.740821087 | 3.09076E-37 | Down |
| 95281 | MFS permease | 1.360771669 | 2.91834E-09 | Up |
| 68698 | Synaptic vesicle transporter SVOP | 0.928518259 | 0.15423063 | NS |
| 95062 | Galactose permease | -3.679314193 | 1.01575E-10 | Down |
| 137795 | MFS transporter | -0.170585539 | 0.245106624 | NS |
| 91910 | Fe^2+^/Zn^2+^ regulated transporter | -4.292743317 | 3.71539E-35 | Down |
| 132334 | Monocarboxylate transporter | -0.162188214 | 0.810598368 | NS |
| 90255 | Synaptic vesicle transporter SVOP | 0.83829123 | 0.840695573 | NS |
| 121436 | Synaptic vesicle transporter SVOP | -0.30452894 | 0.00942677 | Down |
| 128552 | Sugar transporter | -1.605831438 | 0.004358532 | Down |
| 104377 | Peptide transporter | 1.168131873 | 0.005019351 | Up |
| 76562 | Sugar transporter | -0.643925007 | 0.028886471 | Down |
| 139005 | Cation transporting ATPase | -0.551365481 | 0.081700165 | NS |
| 75459 | ABC transporter | -0.052509649 | 0.899287135 | NS |
| 33912 | MDR-type ABC transporters | -0.246188872 | 0.063481651 | NS |
| 138024 | ABC transporter | 0.541611738 | 0.058534043 | NS |
| 142607 | ABC transporter | -0.671370221 | 0.000105954 | Down |
| 91065 | Phosphate transporter | 2.876276593 | 0.604932622 | NS |
| 25083 | Acetyl-CoA carboxylase | -0.778007711 | 0.000834524 | Down |
| 5737 | Cd^2+^/Zn^2+^ transporters | -2.18670992 | 0.05967095 | NS |
| 74437 | Sugar transporter | -0.919081965 | 0.00448989 | Down |
| 26904 | H^+^/oligopeptide symporter | -0.330423855 | 0.462180348 | NS |
| 97054 | ABC transporter | 0.462865213 | 0.0001939 | Up |
| 127980 | MFS lactose permease | 2.713643615 | 4.59904E-87 | Up |
| 136988 | Sugar transporter | 1.183290927 | 3.40981E-14 | Up |
| 131737 | Sugar transporter | 0.945292524 | 0.174396675 | NS |
| 115870 | MFS general substrate transporter | 0.187596915 | 0.584163646 | NS |
| 45579 | MFS permease | -2.864827863 | 0.017103585 | Down |
| 86271 | Amino acid transporters | -1.620128911 | 0.000848545 | Down |
