# Supplementary material for: Mechanism of Zn2+ regulation of cellulase production in Trichoderma reesei Rut-C30
Source: Biotechnol Biofuels Bioprod. 2023 Apr 28;16:73. doi: 10.1186/s13068-023-02323-1 (PMC10148476; doi:10.1186/s13068-023-02323-1)
Supplement: Supplementary file 10 — Additional file 10: Table S4. Primers used in this study. [file 13068_2023_2323_MOESM10_ESM.docx]

**Table S4** Primers used in this study.

| Primer | oligos Sequences (5’ to 3’) |
| --- | --- |
| **Construction of *plc-e* gene deletion vector** | |
| *plc-e*-D1 | ATTACGAATTCTTAATTAACGCTCTTTCTTGTCGCCTAAA |
| *plc-e*-D2 | CATTATACGAAGTTATTCTAGAGATGATATACGCTTCGCTTCCA |
| *plc-e*-D3 | ACTAGTGAGCTCATTTAGGCGTGATATCTCGACAATCT |
| *plc-e*-D4 | AGTGCCAAGCTTATTTTTATTCTAGCACTTGGCGGTTG |
| D70-4 | TCGGACTTGCGGAGGATGTTGTAT |
| Hg3.6 | TGCCTAGTGAATGCTCCGTAACA |
| **Verification of the *plc-e* gene deletion mutants** | |
| *plc-e*-CF | CGGCGTTCTGGTCTAGTAGCA |
| *plc-e*-CR | TCACGATGATGACGACTCAGCA |
| *plc-e*-OF | ACTGACCAACGAGACCGACTAC |
| *plc-e*-OR | GGGAGCCACGATGTAAGAGACT |
| **Construction of *zafA* gene deletion vector** | |
| *zafA*-D1 | ATTACGAATTCTTAATTAATGAGATCCGTCTGTCGAGAAC |
| *zafA*-D2 | CATTATACGAAGTTATTCTAGAGCAGAAGGAAAGGGAAAGTGG |
| *zafA*-D3 | ACTAGTGAGCTCATTTCATACGACCGCTGGCATTAC |
| *zafA*-D4 | AGTGCCAAGCTTATTTGGCTGATGTATGTTGGGCTTAA |
| **Verification of the *zafA* gene deletion mutants** | |
| *zafA*-CF | GTGCTTGGTTGTGGAGGTTCTG |
| *zafA*-CR | CGTCACCCATCTCGGCAAGA |
| *zafA*-OF | CGCAAGCTCGACATGGCAAT |
| *zafA*-OR | AAGGGCACACTTTGGGATGGT |
| **Construction of *crz1* gene deletion vector** | |
| *crz1*-D1 | ATTACGAATTCTTAATTAACTGCCTCCTCCATCATCATCA |
| *crz1*-D2 | CATTATACGAAGTTATTCTAGATGTCTCCGTACCTCGTTGTC |
| *crz1*-D3 | ACTAGTGAGCTCATTTTTCCACACCTAGCTGTCTTCTT |
| *crz1*-D4 | AGTGCCAAGCTTATTTGTTCTCAATTACGGCAGTCCTT |
| **Verification of the *crz1* gene deletion mutants** | |
| *crz1*-CF | GCTGCTAGAAGACCACCTACCT |
| *crz1*-CR | ACGCAATTACGGACCACGACT |
| *crz1*-OF | TCGTCGCCGCCAACTCATT |
| *crz1*-OR | CGCAAGATCAACGCCGAGAG |
| **Quantitative RT-PCR analysis** | |
| Q*sar1*-1 | TGGATCGTCAACTGGTTCTACGA |
| Q*sar1*-2 | GCATGTGTAGCAACGTGGTCTTT |
| Q*cbh1*-1 | CTCCATCTCCGAGGCTCTTACC |
| Q*cbh1*-2 | GCAAGTGCCGCCATATCTGTTAT |
| Q*cbh2*-1 | GCATATTACGCCTCTGAAGTTAGCA |
| Q*cbh2*-2 | GCATAGTTACCGCCATTCTTGTTG |
| Q*egl1*-1 | GCAGCCTCACCATGAACCAGTA |
| Q*egl1*-2 | CACCGTCAGAGTCCAGGAGATAC |
| Q*egl2*-1 | TGAACAAGTCCGTGGCTCCAT |
| Q*egl2*-2 | ACAATTCGTAGGTCCGCTCCAA |
| Q*xyn1*-1 | GGTTGGACGACTGGATCT |
| Q*xyn1*-2 | GGTTGTCCTCCATGATGTAG |
| Q*xyr1*-1 | CTTCCTCCTCCTGCTCATCG |
| Q*xyr1*-2 | TCGTGTGCCCTAACAATGGTC |
| Q*ace3*-1 | GCCAAGTGCGAGTACCTCAG |
| Q*ace3*-2 | GCTGGTCGCTCTTCTTCCTC |
| Q*cam*-1 | AACAACGGCTCCATCGACTT |
| Q*cam*-2 | GTTGTTGTCGCGGTCAAAGA |
| Qc*na1*-1 | CGTGTGACGGAGCTCAAGAC |
| Q*cna1*-2 | GATGCTCGTTCTGCAAGTCG |
| Qc*rz1*-1 | CCAGCAGATGCCGGACACCA |
| Q*crz1*-2 | GTGCATATCGCCGCCCATGC |
| Q*sod1*-1 | CCTCTGCCGGCCCTCACTTC |
| Q*sod1*-2 | AATGGTGCCCTTGGCGTTGC |
| Q*cat1*-1 | GTGCGGCAGAGACCCGGATT |
| Q*cat1*-1 | TCGGGATCTGCCTGGTCGGT |
