# Supplementary material for: Mechanism of Zn2+ regulation of cellulase production in Trichoderma reesei Rut-C30
Source: Biotechnol Biofuels Bioprod. 2023 Apr 28;16:73. doi: 10.1186/s13068-023-02323-1 (PMC10148476; doi:10.1186/s13068-023-02323-1)
Supplement: Supplementary file 11 — Additional file 11: Figure S7. Construction and verification of T. reesei deletion mutant, which were performed as described in our previous study [1]. a: The skeleton schematic diagram to delete plc-e in the parent strain RUT-C30. b: The skeleton schematic diagram to delete zafA in the parent strain RUT-C30. c: The skeleton schematic diagram to delete crz1 in the parent strain RUT-C30. d: Validated electrophoretic diagram to verify the knockout of plc-e, zafA, and crz1 in the parent strain RUT-C30, respectively; M: marker. [file 13068_2023_2323_MOESM11_ESM.docx]

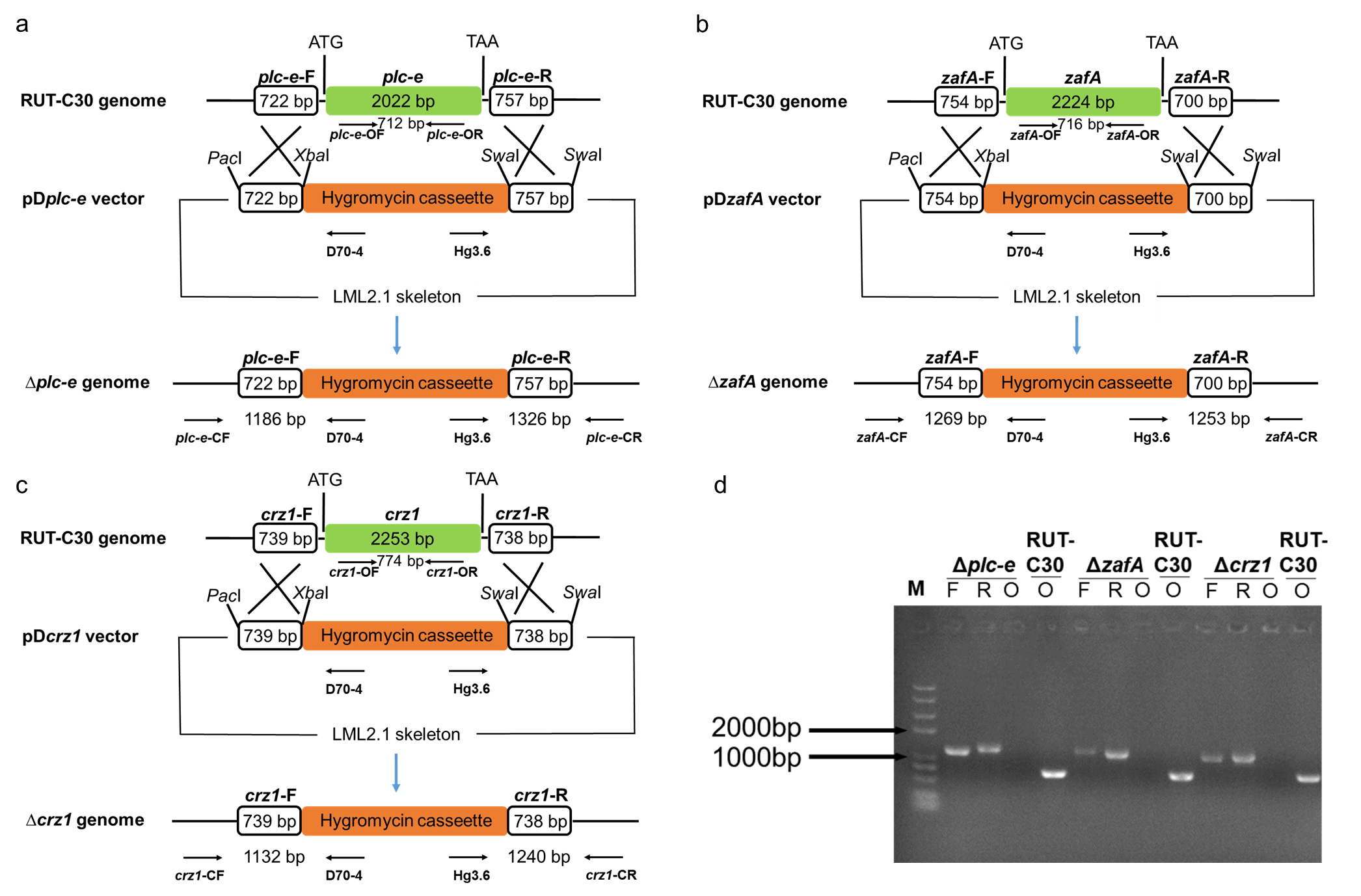


**Fig. S7** Construction and verification of *T. reesei* deletion mutant, which were performed as described in our previous study [1]. **a**: The skeleton schematic diagram to delete *plc-e* in the parent strain RUT-C30. **b**: The skeleton schematic diagram to delete *zafA* in the parent strain RUT-C30. **c**: The skeleton schematic diagram to delete *crz1* in the parent strain RUT-C30. **d**: Validated electrophoretic diagram to verify the knockout of *plc-e*, *zafA*, and *crz1* in the parent strain RUT-C30, respectively; M: marker.

**References**

1. Li N, Zeng Y, Chen YM, Shen YL, Wang W. Induction of cellulase production by Sr^2+^ in *Trichoderma reesei* via calcium signaling transduction. Bioresour Bioprocess. 2022;9:96.
